# Assessing Emotional Intelligence Abilities, Acquiescent and Extreme Responding in Situational Judgment Tests Using Principal Component Metrics

**DOI:** 10.3389/fpsyg.2022.813540

**Published:** 2022-04-26

**Authors:** Johnny R. J. Fontaine, Eva K. Sekwena, Elke Veirman, Katja Schlegel, Carolyn MacCann, Richard D. Roberts, Klaus R. Scherer

**Affiliations:** ^1^Department of Work, Organization and Society, Faculty of Psychology and Educational Science, Ghent University, Ghent, Belgium; ^2^Faculty of Economic and Management Sciences, North West University, Potchefstroom, South Africa; ^3^Department of Experimental Clinical and Health Psychology, Faculty of Psychology and Educational Science, Ghent University, Ghent, Belgium; ^4^Department of Internal Medicine and Pediatrics, Faculty of Medicine and Health Sciences, Ghent University, Ghent, Belgium; ^5^Department of Personality, Differential Psychology and Assessment, University of Bern, Bern, Switzerland; ^6^School of Psychology, The University of Sydney, Sydney, NSW, Australia; ^7^RAD Science Solution, Philadelphia, PA, United States; ^8^Swiss Center for Affective Sciences, University of Geneva, Geneva, Switzerland; ^9^Department of Psychology, University of Munich, Munich, Germany

**Keywords:** ability emotional intelligence, acquiescent responding, extreme responding, principal component metrics, internal structure, nomological network

## Abstract

Principal Component Metrics is a novel theoretically-based and data-driven methodology that enables the evaluation of the internal structure at item level of maximum emotional intelligence tests. This method disentangles interindividual differences in emotional ability from acquiescent and extreme responding. Principal Component Metrics are applied to existing (Mayer-Salovey-Caruso Emotional Intelligence Test) and assembled (specifically, the Situational Test of Emotion Understanding, the Situational Test of Emotion Management, and the Geneva Emotion Recognition Test) emotional intelligence test batteries in an analysis of three samples (total *N* = 2,303 participants). In undertaking these analyses important aspects of the nomological network of emotional intelligence, acquiescent, and extreme responding are investigated. The current study adds a central piece of empirical validity evidence to the emotional intelligence domain. In the three different samples, theoretically predicted internal structures at item level were found using raw item scores. The validity of the indicators for emotional intelligence, acquiescent, and extreme responding was confirmed by their relationships across emotional intelligence tests and by their nomological networks. The current findings contribute to evaluating the efficacy of the emotional intelligence construct as well as the validity evidence surrounding the instruments that are currently designed for its assessment, in the process opening new perspectives for analyzing existing and constructing new emotional intelligence tests.

## Introduction

Evidence for the efficacy of maximum emotional intelligence (EI) as a set of related cognitive abilities that work with emotions mainly comes from studies on the internal structure across EI ability tests and the nomological network of EI scores. Confirmatory factor analyses have repeatedly identified a single higher-order factor across tests for EI abilities (e.g., Schulze and Roberts, 2005). Furthermore, expected large correlations have been observed with traditional intelligence tests (e.g., MacCann, 2010; MacCann et al., 2014), and expected small to moderate relationships with personality (e.g., Rivers et al., 2007), psychopathology (e.g., Matthews et al., 2006), well-being (e.g., Salovey et al., 2000), and self-reported (i.e., typical) EI (e.g., Brackett et al., 2006).

Despite cumulative validity evidence about the internal structure across EI ability tests and about the nomological network, there is one central type of validity evidence that is largely lacking. Demonstrating that the internal structure of an instrument at item level corresponds with the theoretically expected structure is a key source of validity evidence both conceptually (e.g., Messick, 1989; Borsboom et al., 2004) and in terms of normative standards (e.g., Standard 1.13 of Standards for Educational and Psychological Testing, American Educational Research Association et al., 2014). When it comes to EI tests, it is assumed that each EI ability (sub)test assesses a specific unidimensional EI ability construct. For instance, according to the most well-known model in the EI domain, the four-branch model of Mayer and Salovey, there are four specific EI abilities that are expected to be mutually correlated and give rise to one higher-order emotional intelligence factor (e.g., Mayer et al., 2008). In the Mayer Caruso Salovey Emotional Intelligence Test (MSCEIT, Mayer et al., 2002), which operationalizes this four-branch model, there are two subtests per branch that aim to assess the specific unidimensional EI ability. The eight subtests are supposed to jointly assess general emotional intelligence. However, very few studies have investigated the internal structure of EI ability tests, such as the MSCEIT, at item level, and when they do mixed evidence is often found (e.g., Fiori et al., 2014). While some studies report a unidimensional structure, especially studies that apply IRT modeling (Allen et al., 2014; Allen et al., 2015), other studies report structures that are difficult to interpret or are inconsistent (Gignac, 2005; Føllesdal and Hagtvet, 2009; Austin, 2010). For those studies that do report a well-fitting structure, this structure is often obtained after removing non-fitting items, and it is not clear why the discarded items do not fit the hypothetical factor structure (e.g., Palmer et al., 2005).

A likely cause for these inconsistent findings is the use of consensus proportion scoring on Likert-type responses. Most EI ability tests work with Likert-type response scales to evaluate the correctness of items. With consensus proportion scoring each raw response is scored by the proportion of participants (ideally in a representative reference sample) or of experts that have selected that response as the correct response for the item (e.g., Mayer et al., 1999). Recently Legree et al. (2014) criticized consensus proportion scoring due to its sensitivity to irrelevant response characteristics. With Likert-type EI response scales three characteristics of the responses across items can be identified: the shape, the elevation, and the scatter of the response profile. According to Legree et al. (2014), only the shape of the response profile contains meaningful information about the maximum EI construct. These authors consider both elevation and scatter as response characteristics that differ between individuals, but are irrelevant to the ability construct the test intends to measure. Since score elevation and score scatter affect consensus proportion scores, they both introduce bias in the measurement of emotional abilities. This could account for the inconsistent internal structures observed with consensus proportion scores frequently found in the literature.

To overcome the biasing effect of individual differences in score elevation and score scatter, Legree et al. (2014) proposed scoring EI ability as the similarity between the observed and the correct response profile across all items in the ability test (typically by computing a Pearson correlation). However, while profile similarities are insensitive to individual differences in score elevation and score scatter, they do not provide any information about the possible uni- or multi-dimensionality of the internal structure. Even when an EI ability (sub)test assesses various related, but clearly differentiated EI abilities, profile similarities may be computed. Moreover, these profile similarities can display consistent relationships across EI ability tests as well as meaningful nomological networks. This means that the claim that EI ability tests each assess a unidimensional EI ability is currently indeterminate, in turn casting doubt on the construct of general emotional intelligence.

### EI Abilities, Acquiescent Responding, Extreme Responding and Personality Traits

The current approach started by identifying the theoretical constructs that could account for the observed responses on EI items. Based on the existing methodological literature on EI and Likert-scales, four theoretical constructs were identified that could account for the raw item responses in ability tests: (1) the specific EI ability a test was designed to measure, (2) acquiescent responding, (3) extreme responding, and (4) general personality traits and specific preferences. The expected effects of these constructs on the EI item scores, on the correlations between EI items, as well as on the principal component structure that emerges from observed correlations was examined.

#### EI Ability

In existing EI ability tests, a single score is computed per test under the assumption that it assesses a unidimensional ability construct. For instance, it is assumed that the Faces test of the MSCEIT assesses the unidimensional ability to perceive emotions in facial expressions (Mayer et al., 2002). Since items in EI tests using Likert scales vary *a priori* with respect to how incorrect or correct they are, it can be expected that the higher the EI ability of a participant, the more the participant will rate incorrect items on the lower side of the rating scale and correct items on the upper side of the rating scale. This effect should be more pronounced the more correct or incorrect the item is. As a result, correct items should be mutually positively correlated (and the correlations should be more positive the more correct the items are), incorrect items should be mutually positively correlated (and the correlations should be more positive the more incorrect the items are), and correct and incorrect items should be negatively correlated (and the correlations should be more negative the more one item is correct and the more the other item is incorrect). When a principal component analysis is applied to such a correlational pattern, a bipolar principal component should emerge with correct items having a positive loading (with loadings being more positive the more correct the item is) and incorrect items having a negative loading (with loadings being more negative the more incorrect the item is).

**FIGURE 1:**
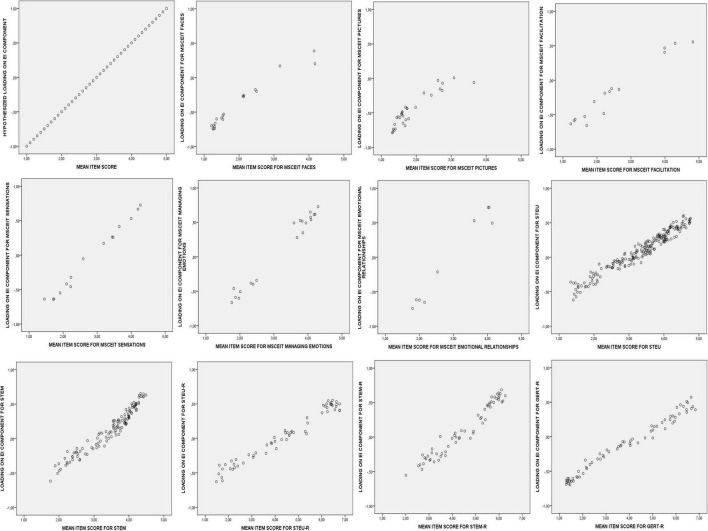
Hypothetical and observed plots of the mean item scores and the item loadings on the bipolar EI component for the 11 EI tests.

It has been demonstrated that the mean item scores in representative samples of lay persons evaluating the correctness of EI items converges with the mean item scores of experts evaluating their correctness (see Mayer et al., 2002; Sanchez-Garcia et al., 2016). This finding implies that the mean item scores in representative samples can be used to identify the extent items are correct (or incorrect). This research also implies that the mean item scores can be used to predict how items should load on the bipolar principal component. The more an item mean is towards the upper end of the response scale, the more correct the item is, and the more positive the item loading on the bipolar component should be within this measurement framework. The more an item mean is to the lower end of the response scale, the more incorrect the item is, and the more negative the item loading on the bipolar component should be within this same framework. Across items in an EI ability test, this means that the profile of loadings on the bipolar component should mirror the profile of mean item scores in a consensus sample (see Panel 1 of Figure 1 for such a prediction). Thus, the first and most central hypothesis (referred to as Hypothesis 1.1 in the current paper) is that EI ability will produce a bipolar principal component that mirrors the mean item scores.

#### Acquiescent Responding

Likert-type response scales are known to be susceptible to acquiescent responding (e.g., Baumgartner and Steenkamp, 2001). Acquiescent responding (and its opposite disacquiescent responding) is the participant’s tendency to systematically agree (or disagree) with an item (or in the context of EI tests to consider an item as more correct or incorrect) independent of the item content. As such, this confounding variable will affect the item scores of a participant in the same direction (e.g., Greenleaf, 1992a; Weijters et al., 2010). In the context of EI assessment, this means that Likert response scales elicit systematic interindividual differences in the tendency to score an EI item as correct or effective irrespective of the content of the items. Acquiescent responding raises the correlations among all items in a test, i.e., positive correlations will become more positive and negative correlations will become less negative (e.g., Russell and Carroll, 1999). In a principal component analysis acquiescent responding should lead to a unipolar component with all items loading positively on it. In the context of maximum EI, it cannot be expected that acquiescent responding will affect all items to the same extent. There is a strong relationship between the item mean, which reflects incorrectness (or correctness) of the item, and the item distribution when using Likert-type scales. Thus, the more an item is incorrect, the lower its score and the more positively skewed the responses will be (i.e., in the case where most respondents choose the lower end of the response scale). And the more an item is correct, the higher the item mean, and the more negatively skewed the item distribution will be (i.e., in this case, most respondents choose the upper end of the response scale). Collectively, this suggests that the extent acquiescent responding affects the item responses depends on the item mean. For items that are either clearly wrong or clearly right, the extreme response options have a very high probability to be selected anyway, and there will be little room for acquiescent responding to have an additional impact. It can thus be expected that the impact of acquiescent responding will be lowest for items that are either clearly incorrect or clearly correct, and highest for items that are neither incorrect, nor correct.

**FIGURE 2:**
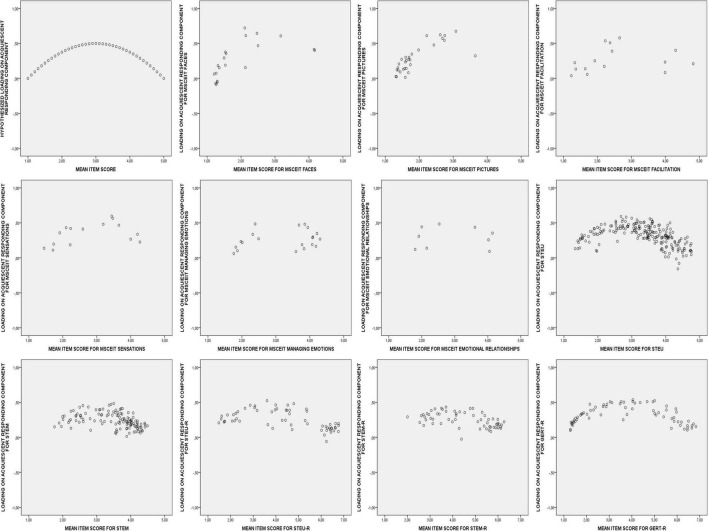
Hypothetical and observed plots of the mean item scores and the item loadings on the unipolar acquiescent responding component for the 11 EI tests.

Since the item mean contains information about correctness in a representative sample, the profile of loadings on the acquiescent responding component can also be precisely predicted. Thus, while all loadings are predicted to be non-negative, the more the item mean is towards the middle of the response scale the more positive its predicted loading on the acquiescent responding component should be (see Figure 2 for an example). Thus, the second hypothesis (referred to as Hypothesis 1.2 in the current paper) is that acquiescent responding will produce a unipolar component with all items having a non-negative loading and with their loading being more positive the more the item mean is towards the middle of the response scale.

#### Extreme Responding

Next to eliciting interindividual differences in scoring EI items as correct (or incorrect) independent of their content, Likert-type rating scales are also vulnerable to extreme responding (or its opposite midpoint responding). Extreme responding allows that participants differ in their preference for more extreme (as opposed to more neutral) responses irrespective of the item content (e.g., Greenleaf, 1992b; Baumgartner and Steenkamp, 2001). In the case of EI tests, this means there will be systematic individual differences in the tendency to score items as either clearly correct or clearly incorrect, rather than using scores in the middle of the response scale. Interindividual differences in extreme responding do not systematically affect the pattern of correlations between items and will thus not emerge as a separate component in a principal component analysis. Extreme responding is often operationalized as the proportion of extreme responses across all items in a test (e.g., Greenleaf, 1992b). However, in the context of maximum EI the observed proportion of extreme responses cannot function as a pure indicator of extreme responding. The more (in)correct an EI item is, the more likely it will be that participants high in EI ability will select either the lowest (in case of an incorrect item) or the highest (in case of a correct item) response option. Thus, the third hypothesis (referred to as Hypothesis 1.3 in this paper) is that the observed proportion of extreme responses is not only produced by interindividual differences in extreme responding, but also by EI ability.

#### Personality

Although EI ability tests are developed to assess a person’s ability in processing emotional information, it cannot be excluded that evaluations of correctness are also influenced by general personality traits or more context-specific preferences. For instance, introverts might systematically consider social withdrawal reactions as being more helpful to manage emotions than extroverts, not only for themselves, but in general. Or people might react differently to situations they feel particularly passionate about (such as leisure activities) compared to other situations. It thus seems plausible that the hypothesized bipolar EI component and unipolar acquiescent responding component will not fully account for the correlational pattern, because (a subset of) items are affected by more general personality characteristics or context-specific preferences.

EI ability tests contain items from a broad range of emotional experiences (e.g., from very different situational antecedents that elicit emotions) often developed on the basis of a situational judgment methodology (e.g., Situational Test of Emotion Management), which implies that many general personality characteristics and specific preferences could potentially affect evaluations of correctness. Therefore, it is impossible to make precise *a priori* predictions about which personality constructs will affect which items in an EI ability test. Still, in well-designed EI tests only EI ability and acquiescent responding should be the major constructs systematically accounting for item correlations, and should thus emerge as the first two principal components.

Since in the current approach there is no claim that EI ability and acquiescent responding are the only constructs that account for the observed responses and thus the correlations between EI items, Principal Component Analysis (PCA) was chosen to analyze the internal structure. PCA, which basically is a reduction technique, is used to investigate whether the first two principal components represent bipolar EI ability and unipolar acquiescent responding. Thus, the fourth hypothesis is that the first two components in a PCA on EI ability items represent EI ability and acquiescent responding (referred to as Hypothesis 1.4 in the remainder of the paper).

#### Principal Component Metrics

If the above predictions hold (a bipolar EI ability component, a unipolar acquiescent responding component, positive correlations between EI ability and the proportion of extreme responses), it is possible to derive scores for EI ability, acquiescent responding, and extreme responding from a PCA on the raw data and the observed proportion of extreme responses. As PCA is a reduction technique, it allows the researcher to compute scores for each principal component as a linear combination of the raw item scores. The principal component scores of the bipolar EI component assess EI ability. In the remainder of the manuscript these component scores will be referred to as the Emotional Intelligence Scores. The principal component scores of the unipolar acquiescent responding component represent acquiescent responding. They will be referred to as the Acquiescent Responding Scores. Finally, the proportion of extreme responses partialled out for the Emotional Intelligence Scores and Acquiescent Responding Scores represent the individual differences in extreme responding. They will be referred to as the Extreme Responding Scores. Since the Emotional Intelligence Scores, Acquiescent Responding Scores, and Extreme Responding Scores are computed or derived from PCA, they are further referred to as the Principal Component Metrics (PCM’s).

### Internal Structures of Emotional Intelligence Scores, Acquiescent Responding Scores, and Extreme Responding Scores Across EI Ability Tests

The contribution of the Principal Component Metrics is that the internal structure is identified at item level for each EI ability (sub)test separately. An important question is now how these Principal Component Metrics relate to one another across EI tests in a test battery.

#### Internal Structure of Emotional Intelligence Scores

If the predicted bipolar components in each of the EI tests are assessing abilities, it is expected that these bipolar components are substantially correlated and can be represented by a single, general factor across EI tests (e.g., Mayer et al., 2008). Thus, substantial positive correlations between Emotional Intelligence Scores from different EI tests that can be represented by a unifactorial model are predicted (Hypothesis 2.1).

#### Internal Structure of Acquiescent Responding Scores

There is substantial evidence that acquiescent responding can be considered as a stable personality trait. Acquiescent responding has been found to be substantially correlated across different psychological tests (e.g., Weijters et al., 2010) and to be stable across time, even with a time interval of eight years (e.g., Wetzel et al., 2015). Therefore, the prediction is that there will be substantial positive correlations between Acquiescent Responding Scores from different EI tests that can be represented by a unifactorial model (Hypothesis 2.2).

#### Internal Structure of Extreme Responding Scores

For extreme responding there is also substantial evidence that it can be considered as a stable personality trait. Extreme responding across psychological tests is positively correlated within the same study (e.g., Weijters et al., 2010) and stable across time (e.g., Wetzel et al., 2015). Therefore, substantial positive correlations between Extreme Responding Scores from different EI tests that can be represented by a unifactorial model are expected (Hypothesis 2.3).

### Nomological Networks of Principal Component Metrics

#### Nomological Network of Emotional Intelligence Scores

If emotional intelligence scores offer a good indicator of EI, the strongest correlations should be observed with traditional intelligence tests and small to medium correlations with self-report EI tests (e.g., MacCann, 2010; Schlegel et al., 2019), small to medium correlations with personality traits (e.g., Van Rooy et al., 2005; Rivers et al., 2007), and zero to small correlations with tests for psychopathology and well-being (e.g., Salovey et al., 2000) (Hypothesis 3.1).

#### Nomological Network of Acquiescent Responding Scores

Acquiescent responding as a personality characteristic is shown to be associated with lack of self-confidence, self-esteem, and assertiveness (Meisenberg and Williams, 2008), and with agreeableness (Graziano and Tobin, 2002; He and van de Vijver, 2013). A negative association is observed with education both at the individual and country level (Meisenberg and Williams, 2008). In line with this finding, a negative correlation has also been observed between acquiescent responding and cognitive ability (Meisenberg and Williams, 2008; Lechner and Rammstedt, 2015). Based on previous research, it is thus expected that acquiescent responding will relate positively to neuroticism and agreeableness, and negatively to cognitive ability (Hypothesis 3.2).

#### Nomological Network of Extreme Responding Scores

There is empirical evidence of a positive relationship between extreme responding and intolerance of ambiguity and decisiveness (e.g., Naemi et al., 2009). In line with this finding, extreme responding has been shown to be positively and significantly related to extraversion (e.g., Austin et al., 2006; He and van de Vijver, 2013) and conscientiousness (e.g., Austin et al., 2006). There seems to be a negative relationship between extreme responding and both educational level and cognitive ability (Meisenberg and Williams, 2008). Based on previous research, it is expected that extreme responding is positively related to extraversion and conscientiousness, and negatively related to cognitive abilities (Hypothesis 3.3).

### Relationships Between Profile Similarity Metrics and Principal Component Metrics

Based on the theoretical expectations underlying the Principal Component Metrics (PCM) straightforward hypotheses can be formulated about how they relate to the Profile Similarity Metrics (PSM). The EI ability is predicted to cause both the bipolar EI factor and the similarity between the observed and the correct profile of scores across items in an EI test. Therefore, PCM Emotional Intelligence Scores and PSM Shape are predicted to be highly correlated (Hypothesis 4.1). Since acquiescent responding implies that all items are scored systematically higher (or lower), acquiescent responding should lead to an increase (or decrease) of score elevation. There should thus be a high correlation between PCM Acquiescent Responding Scores and PSM Elevation (Hypothesis 4.2). For score scatter, the predictions are more complex. Extreme responding implies that participants tend to use extreme scale scores and thus will show more scatter of responses across items in an EI subtest. However, extreme responding is not the only construct that can be expected to affect the scatter of responses across items. For items that are clearly correct (or incorrect), the model predicts that participants that are high in EI ability will score correct items more at the upper end and incorrect items more at the lower end of the response scale. Thus, the model predicts that EI-ability will lead to more scatter across items, especially due to those items that are clearly correct or incorrect. Thus, the prediction is that both PCM Extreme Responding Scores and PCM Emotional Intelligence Scores will be correlated with PSM Scatter (Hypothesis 4.3).

## Materials and Methods

### Participants and Procedure

#### Sample 1

The first sample is a United States student sample. The data was collected under Army Research Institute funding to develop and validate alternative measures of EI (Roberts et al., 2012). These data have been previously analyzed using conventional scores (MacCann et al., 2014) and profile similarities (Legree et al., 2014). For the current study, all participants that either had more than 20% of missing values on all of the six MSCEIT EI tests with a rating response format or systematically used the same response option on each of these six MSCEIT EI tests were first removed from the dataset (which was 1.8% of the data). Of the remaining 713 students, 225 enrolled at a two-year college and 488 at a four-year college. The overall age range was 17 to 59 with a mean age of 21.7 years and a standard deviation of 5.98. Of these participants 287 were male and 426 were female. Reported ethnicities were as follows: 64.2% White; 15.6% African American; 10.4% Hispanic; 3.9% Asian; 1.4% American Indian or Alaskan Native; 0.7% Native Hawaiian or other Pacific Islander; and 3.8% Multi-racial.

#### Sample 2

The second sample is a mixed student-adult sample from the Dutch-speaking part of Belgium. Students were asked to invite two participants to participate in an anonymous web-based survey as part of their assignment for a psychological assessment course. All participants that used the same response category for more than 80% of the items on either the STEU or the STEM were removed from the dataset (1.8%). Of the remaining 828 participants, 417 were females and 411 males; and 129 were students. With respect to educational level, 91 had less than secondary education, 467 successfully finished secondary education, and 270 held at least a bachelor’s degree. The age ranged from 16 to 60 with a mean age of 38.4 years and a standard deviation of 12.52.

#### Sample 3

The third sample is also a mixed student-adult sample from the Dutch-speaking part of Belgium. Students were asked to invite one participant to participate in an anonymous web-based survey as part of their assignment for a course on psychological assessment. They themselves were also invited to participate in the research on a completely free and anonymous basis. All participants that used the same response category for more than 80% of the items for either the STEU-R, the STEM-R, or the GERT-R were removed from the data base (2%). Of the remaining 762 participants, 426 were females and 336 males and 54 were regular students. With respect to educational level, 78 had less than secondary education, 356 successfully finished secondary education, and 328 held at least a bachelors’ degree. The age ranged from 18 to 78 with a mean age of 35.7 years and a standard deviation of 12.78.

### Instruments

#### Sample 1

Mayer-Salovey-Caruso EI Test (MSCEIT; Mayer et al., 2003). The MSCEIT assesses four hierarchically ordered branches of emotion-related abilities: (a) perception of emotions (measured by faces and pictures tasks); (b) facilitation of emotions into thought processes (measured by sensations and facilitation tasks); (c) understanding of emotions (measured by blends and changes tasks); and (d) management of emotions (measured by emotion management and emotional relationships tasks). Since the two EI tests for emotion understanding (blends and changes tasks) used a multiple-choice format, they were not included in the current study. The other six EI tests of the MSCEIT used a five-point Likert scale to rate the correctness or effectiveness of the items.

Assessment of intelligence. In total 15 computerized tests from the ETS Factor Reference Kit and from retired items of the testing programs (with the permission of ETS, MacCann et al., 2014, p. 364) with markers for Gf, Gc, Gv, Gr, and Gq (i.e., fluid, crystallized, visual, retrieval and quantitative abilities) where applied.

Assessment of personality. The personality items were taken from the International Personality Item Pool (Goldberg et al., 2006), and were selected to measure openness (111 items), conscientiousness (113 items), extraversion (81 items), agreeableness (102 items), and neuroticism (118 items). Participants rated their agreement with each item on a five-point scale from “Not at all like me” (1) to “Very much like me” (5).

Trait EI questionnaire-short form (TEIQue-SF). The TEIQue-SF (Petrides and Furnham, 2001) is a self-report EI scale that consists of 30 items measuring global trait EI (trait EI). Participants rated their agreement with each item on a 7-point scale from “Completely Disagree” (1) to “Completely Agree” (7).

#### Sample 2

The Situational Test of Emotional understanding (STEU) (MacCann and Roberts, 2008). The STEU assesses emotional understanding of own and other’s emotions. In the standard version this scale consists of 42 multiple-choice item stems with 5 possible reactions. In the current study, respondents were asked to rate each of the MC responses of all item stems on a five-point Likert scale (completely disagree, disagree, nor disagree/neither agree, agree, completely agree). In total there were 210 STEU items (42 item stems × 5 possible reactions to each item stem).

The Situational Test of Emotion Management (STEM) (MacCann and Roberts, 2008). Thirty items were taken from the STEM to assess emotion management (as in Study 2 of MacCann and Roberts, 2008). This version of the STEM uses a five-point rating format (1) very ineffective, (2) ineffective, (3) not ineffective/neither effective, (4) effective, (5) very effective. In total there were 120 STEM items (30 item stems x 4 possible reactions to each item stem).

The Rosenberg Self-Esteem Scale (RSES). The 10 items of the RSES assess a person’s overall evaluation of his or her worthiness as a human being (Rosenberg, 1965). Responses were coded on a 4-point scale ranging from 1 (strongly disagree) to 4 (strongly agree).

Toronto Alexithymia Scale (TAS-20). The TAS-20 (Bagby et al., 1994) consists of 20 self-report items rated on a 5-point scale from strongly disagree (1) to strongly agree (5). It indexes three aspects of alexithymia: (a) difficulty identifying feelings (DIF); (b) difficulty describing feelings (DDF); and (c) an externally oriented thinking style (EOT).

#### Sample 3

Situational Test of Emotional Understanding Reduced form (STEU-R). A short form of the STEU was constructed consisting of 60 items: 30 item stems and two reactions per item stem. The items were selected in such a way that they varied from highly improbable to highly probable on the basis of the empirical data from the previous sample with the STEU. The items of the STEU-R were scored on a seven-point Likert scale (1) very unlikely, (2) unlikely, (3) rather unlikely, (4) nor unlikely/neither likely, (5) rather likely, (6) likely, (7) very likely. For instance, the example item is reformulated as follows: “Clara receives a gift. A. How likely is it that Clara feels happy? B. How likely is that Clara feels bored?” The participants than had to rate both emotions on the 7-point Likert scale.

Situational Test of Emotional Management Reduced form (STEM-R). A short form of the STEM was constructed consisting of 60 items (30 item stems and two reactions per item stem). The items were selected in such a way that they varied from highly inefficient to highly efficient on the basis of the empirical data from the previous sample with the STEM. The items of the STEM-R were scored on a seven-point Likert scale (1) very ineffective, (2) ineffective, (3) rather ineffective, (4) nor ineffective/neither effective, (5) rather effective, (6) effective, (7) very effective. For instance, the following item was used as an example item: “The woman who relieves Celia at the end of her shift is twenty minutes late without excuse or apology. How effective are the following actions for Celia? A. To call her names. B. Tell the boss about it.” The participants then had to rate both reactions on the 7-point Likert scale.

Geneva Emotional Recognition Test Reduced form (GERT-R). The original GERT is a computer-based test that measures the ability to recognize emotions in others (Schlegel et al., 2014; Schlegel and Scherer, 2015). The GERT consists of 83 short video clips with sound in which ten actors express 14 different emotions: pride, joy, amusement, pleasure, relief, interest, surprise, anxiety, fear, despair, sadness, disgust, irritation, and anger. Participants are asked to choose which of the 14 emotions were expressed by the actor and responses are coded as either correct or incorrect. Based on a previous empirical study in Dutch with the original GERT instrument (Schlegel et al., 2019), 70 items were selected for the present study, each consisting of a multimodal emotional display (i.e., video) and one emotion term that participants were asked to rate. For each of the 14 emotions, five displays (i.e., video clips) were selected. For each of the 70 videos, participants rated how likely one of the 14 emotions was being expressed by the actor on a seven-point Likert scale (1) very unlikely, (2) unlikely, (3) rather unlikely, (4) nor unlikely/neither likely, (5) rather likely, (6) likely, (7) very likely. Each of the 14 emotion terms was used five times in the whole test. The combinations between the emotional display and the emotion terms were selected in such a way that they ranged from highly unlikely to highly likely. For instance, for a video clip in which an actor expressed irritation, the participant was asked how likely it was that the actor expressed disgust on the 7-point Likert scale.

Trait EI questionnaire – short form (TEIQue-SF). See above (Sample 1).

Toronto Alexithymia Scale (TAS-20). See above (Sample 2).

The Rosenberg Self-Esteem Scale (RSES). See above (Sample 2).

HEXACO-60. The HEXACO-60 (Ashton and Lee, 2009) consists of 60 items assessing the six personality factors of the HEXACO model of personality: Honesty-Humility, Emotionality, Extraversion, Agreeableness, Conscientiousness, and Openness to Experience. Each of the six scales consists of 10 items that cover a wide range of content. All items employ a 1 (strongly disagree) to 5 (strongly agree) response scale.

Kaufman Adult Intelligence Scale (KAIT- Kaufman and Kaufman, 1993). It includes two components, a core battery and an expanded battery. For the current study only the core battery was used. We thus obtained scores for crystallized intelligence (Gc), fluid intelligence (Gf), and the total IQ score (G).

### Ethical Considerations

Sample 1 constitutes a reanalysis of earlier research reported by MacCann et al. (2014), which passed various internal review standards. For sample 2 and 3 the research was executed according to the general ethical protocol of the Faculty of Psychology and Educational Sciences of Ghent University, Belgium.

### Data Analyses

The internal structure of each EI (sub)test was investigated using Principal Component Analysis on the raw (untransformed) item responses. Because there is rotational indeterminacy (any rotation of the axes representing the principal components in a geometrical representation is mathematically equivalent), the observed component structures were orthogonally Procrustes rotated towards the theoretically expected structures.

Theoretically expected structures were constructed for each EI test (see Supplementary Tables 1–11). For the hypothetical loadings on the bipolar EI-component, the observed item means were linearly transformed so that the minimum of the response scale (1 in all three samples) corresponded with a loading of -1, the middle of the response scale (3 in sample 1 and 2, and 4 in sample 3) corresponded with a loading of 0, and the maximum of the response scale (5 in sample 1 and 2, and 7 in sample 3) corresponded with a loading of 1 (see Figure 1, Panel 1). For the hypothetical loadings on the acquiescent responding component the observed means were curvilinearly transformed so that both the minimum (1 in all three samples) and the maximum (5 in sample 1 and 2, and 7 in sample 3) of the response scale corresponded with a loading of 0, and the middle of the response scale (3 in sample 1 and 2, and 4 in sample 3) corresponded with a loading of 0.50 (see Figure 2, Panel 1).

To evaluate how well the observed pattern of component loadings after orthogonal Procrustes rotation confirmed the expected pattern of component loadings, the Tucker’s phi congruence coefficient was computed per component. The Tucker’s phi ranges from 0 (no congruence between the pattern of loadings across items) to 1 (perfect congruence between the pattern of loadings across items). Thus, the closer the Tucker’s phi is to 1 the more the observed pattern of loadings corresponds with the hypothetical pattern of loadings across items. In the literature, Tucker’s phi values of 0.85, 0.90, and 0.95 are considered to point to a fair, a good and a very good congruence, respectively, between the observed and hypothetical loading pattern (Lorenzo-Seva and ten Berge, 2006; Fischer and Fontaine, 2010). The Tucker’s phi congruence coefficients have been computed in SPSS (version 26) using a syntax command, which can be obtained from the first author (Johnny.Fontaine@ugent.be).

## Results

### Internal Structure and Principal Component Metrics of Individual EI Tests

In the two-componential structures of all 11 EI tests, the observed EI component showed a very good congruence with the predicted EI component after orthogonal Procrustes rotation (with Tucker’s phi’s being 0.95 and higher) (see Table 1). The congruence with the second predicted acquiescent responding component was higher than 0.85 for nine of the 11 EI tests. As it is possible that personality traits or specific preferences interfere with the expected EI and acquiescent responding component, we also investigated whether three-componential solutions (and if needed higher-componential solutions) allowed for a better identification of the expected EI and acquiescent responding component. In a three-componential structure there was a substantial increase in the congruence of the acquiescent responding component for the MSCEIT Facilitation (Tucker’s phi raising from 0.52 to 0.90) and the MSCEIT Emotional Relationships (Tucker’s phi raising from 0.40 to 0.92). Moreover, for the MSCEIT Pictures there was also a clear increase in the congruence of the acquiescent responding component (Tucker’s phi raising from 0.88 to 0.95). Because the predicted Acquiescent responding components emerged more clearly in a three- than a two-componential structure for the MSCEIT Facilitation, Emotional Relationships, and Pictures, a three-componential structure was selected for these three EI tests. For the other eight EI tests, a two-componential structure was selected.

**TABLE 1:** Congruence measures (Tucker’s Phi’s) with *a priori* EI and acquiescent responding component loadings after orthogonal Procrustes rotation, number of selected components, and internal consistencies (Cronbach’s Alpha’s) of component scores.

Sample/EI-test	ϕ_*EI*_-2	ϕ_*AC*_-2	ϕ_*EI*_-3	ϕ_*AC*_-3	NC	α_*EIS*_	α_*ARS*_
**Sample 1: MSCEIT**							
Faces	0.99	0.92	0.99	0.92	2	0.86	0.64
Pictures	0.98	0.88	0.98	0.95	3	0.86	0.62
Facilitation	0.95	0.52	0.98	0.90	3	0.70	0.33
Sensations	0.99	0.97	0.99	0.98	2	0.77	0.57
Management	0.99	0.91	0.98	0.92	2	0.85	0.38
Relationships	0.99	0.40	0.98	0.92	3	0.79	0.00
**Sample 2**							
STEU	0.98	0.95	0.97	0.95	2	0.95	0.97
STEM	0.96	0.94	0.95	0.96	2	0.93	0.88
**Sample 3**							
STEU-R	0.98	0.93	0.98	0.93	2	0.89	0.81
STEM-R	0.97	0.95	0.96	0.94	2	0.90	0.79
GERT-R	0.97	0.97	0.97	0.97	2	0.92	0.88

*ϕ_EI_-2: congruence of EI component loadings in two-componential structure after orthogonal Procrustes rotation, ϕ_AC_-2: congruence of AC component loadings in two-componential structure after Procrustes orthogonal rotation, ϕ_EI_-3: congruence of EI component loadings in three-componential structure after orthogonal Procrustes rotation, ϕ_AC_-3: congruence of AC component loadings in three-componential structure after orthogonal Procrustes rotation, NC: selected number of components, α_EIS_: internal consistency of EI component scores, α_ARS_: internal consistency of acquiescent responding component scores.*

#### EI Abilities

Hypothesis 1.1. is fully confirmed. In each EI test a bipolar component is observed that mirrors the mean item scores. Panels 2 to 12 of Figure 1 display the observed component loadings on the first principal component after orthogonal Procrustes rotation plotted against the mean item scores per EI test. These plots visually represent the high congruence (Tucker’s phi) between the predicted and the observed loadings on the EI component (see also Supplementary Tables 1–11 for the item loadings per EI test).

Principal component scores on the first bipolar EI component were computed per EI test to assess maximum EI (further referred to as Emotional Intelligence Scores, EIS), which are a weighted sum of the observed item scores. For each EI test the internal consistency of this weighted sum was computed using Cronbach’s alpha, which ranged from 0.70 to 0.95 across the 11 EI tests (see Table 1).

#### Acquiescent Responding

Hypothesis 1.2., which predicted that there would be a unipolar acquiescent responding component, was also confirmed. Figure 2 panels 2 to 12 display the observed component loadings after orthogonal Procrustes rotation plotted against the mean item scores. These plots visually represent the good congruence between the hypothesized and observed component loadings: (virtually) all loadings are non-negative, with loadings tending to be highest for items with a mean score close to the response scale mean.

Principal component scores on the second unipolar acquiescent responding component were computed per EI test to assess acquiescent responding tendency (further referred to as Acquiescent Responding Scores, ARS). For each EI test, the reliability of this weighted sum was computed using Cronbach’s alpha, which ranged from 0.00 to 0.97. The reliability of the Acquiescent Responding Scores was low for the MSCEIT EI tests. Acquiescent Responding Scores were especially unreliable for the MSCEIT Facilitation, Emotional Management, and Emotional Relationships. The reliability was good to very good in the STEU, STEM, STEU-R, STEM-R, and GERT-R (see also Table 1).

#### Extreme Responding

As predicted by Hypothesis 1.3., the proportion of extreme responses was highly related to the Emotional Intelligence Scores (with correlations ranging from 0.59 to 0.73, see Supplementary Table 12 for the correlation per subtest). These relationships confirm that the observed proportion of extreme responses cannot just be taken as an indicator of extreme responding. Moreover, correlations were observed with the Acquiescent Responding Scores (with correlations ranging from −0.38 to 0.14). Especially in the MSCEIT Faces and MSCEIT Pictures – which are EI tests with very low mean item scores – a moderate negative relationship was observed between the Acquiescent Responding Scores and the proportion of extreme responses. The stronger acquiescent responding, the smaller was the proportion of extreme responses in especially these two EI tests. Extreme responding was therefore scored by the proportion of extreme responses statistically controlled for not only by the Emotional Intelligence Scores, but also the Acquiescent Responding Scores using regression analyses. These scores are in the remainder of the text referred to as Extreme Responding Scores (ERS). Since the Extreme Responding Scores were computed after statistically controlling for the Emotional Intelligence Scores and the Acquiescent Responding Scores no classical reliability coefficient could be computed for this measure of extreme responding.

#### General Personality Traits and Specific Preferences

For the three EI tests for which the congruence of the acquiescent responding component was improved by adding a third principal component, the content of the third component was further investigated (see also Supplementary Tables 2, 3, 6). For MSCEIT Pictures and MSCEIT Facilitation positively valenced items tended to load positively and negatively valenced items tended to load negatively on the third component. Items from one single scenario loaded on the third component of MSCEIT Emotional Relationships, thus pointing to substantial interindividual differences in how people deal with that specific scenario. The scores on these three additional components were uncorrelated, and thus did not point to a shared personality trait. Hypothesis 1.4. was thus only partially confirmed. The bipolar EI component systematically emerged in a two-componential representation. However, to properly identify the unipolar acquiescent responding component, an additional component was needed for three of the 11 EI tests.

### Internal Structures of Emotional Intelligence Scores, Acquiescent Responding Scores, and Extreme Responding Scores Across EI Ability Tests

Correlations were computed to investigate the relationships between the Emotional Intelligence Scores, Acquiescent Responding Scores, and Extreme Responding Scores from different EI tests in each of the respective samples. Moreover, where possible a Confirmatory Factor Analysis (CFA) was executed. Only in the first sample, were there enough EI tests to straightforwardly test the predicted unifactorial CFA model. In the third sample, the predicted unifactorial CFA model could be estimated, but could not be statistically tested as the CFA model was just identified (since there were only three EI tests). In order to still allow for an evaluation of the factor models in the third sample, additional restrictions were imposed. The factor loadings, the intercepts, and error variances of the STEU-R, STEM-R, and GERT-R were held constant for the CFA models in the third sample.

To evaluate the fit of the CFA models the criteria recommended by Schweizer (2010) were followed: a root mean square error of approximation (RSMEA) below 0.05 and a comparative fit index (CFI) higher than 0.95 are considered to indicate good model fit; a RSMEA between 0.05 and 0.08, and a CFI between 0.90 and 0.95 indicate acceptable fit. Moreover, standardized root mean square residuals (SRMR) should be lower than 0.10.

#### Internal Structure of Emotional Intelligence Scores Across EI Tests

In the first sample the correlations between the Emotional Intelligence Scores of different EI tests ranged from 0.34 to 0.68 with an average of 0.46. A CFA with a single factor, the Emotional Intelligence Scores of the six EI tests as indicators, and one error covariance between MSCEIT Emotional Management and MSCEIT Emotional Relationships fitted acceptably: Chi^2^ (8) = 42.97, RMSEA = 0.08, CFI = 0.97, SRMR = 0.03. The error covariance can be well justified based on the design of the test as both EI subtests are supposed to measure managing emotions and are more comparable in terms of content than the other subtests. The Emotional Intelligence Scores of the six EI tests had loadings between 0.57 and 0.81 on the common factor. The composite reliability (McDonald’s coefficient omega) for the common factor was 0.83. In the second sample, the Emotional Intelligence Scores of the STEU and the Emotional Intelligence Scores of the STEM correlated 0.61. In the third sample, the Emotional Intelligence Scores of the three EI tests correlated between 0.55 and 0.57. A confirmatory factor model with the Emotional Intelligence Scores of the three tests as indicators, a single latent factor, and additional equality restrictions on the three factor loadings, the three intercepts, and the three error variances fitted the data well: Chi^2^ (6) = 8.78, RMSEA = 0.03, CFI = 0.99, SRMR = 0.04. The three standardized factor loadings were 0.74. The composite reliability (McDonald’s coefficient omega) for the common factor was 0.78. Thus, in the three samples Hypothesis 2.1 was confirmed.

#### Internal Structure of Acquiescent Responding Scores Across EI Tests

In the first sample the correlations between the Acquiescent Responding Scores of the six EI tests ranged from −0.03 to 0.32 with an average of 0.12. A confirmatory factor model with a single factor (and one error covariance between MSCEIT Emotional Management and MSCEIT Emotional Relationships) fitted well: Chi^2^ (8) = 18.85, RMSEA = 0.05, CFI = 0.95, SRMR = 0.03. The Acquiescent Responding Scores of the six tests had loadings between 0.07 and 0.67 on the common factor. The composite reliability (McDonald’s coefficient omega) for the common factor was 0.42. In the second sample, Acquiescent Responding Scores of the STEU and the STEM correlated 0.50. In the third sample, the Acquiescent Responding Scores of the three tests correlated between 0.22 and 0.32. A confirmatory factor model with the Acquiescent Responding Scores of the three tests as indicators, a single latent factor and additional equality restrictions on the three factor loadings, the three intercepts, and the three error variances fitted the data well: Chi^2^ (6) = 5.96, RMSEA = 0.00, CFI = 1.00, SRMR = 0.03. The three standardized factor loadings were 0.53. The composite reliability (McDonald’s coefficient omega) for the common factor was 0.59. Thus, Hypothesis 2.2. was supported in samples 2 and 3. In sample 1, however, the results were less clear. Although a single factor model fitted the data in the first sample, the loadings were zero to moderate and the composite reliability of the acquiescent responding factor was very low.

#### Internal Structure of Extreme Responding Scores Across EI Tests

In the first sample the correlations between Extreme Responding Scores of the six EI test ranged from 0.08 to 0.37 with an average of 0.21. A confirmatory factor model with a single factor (and one error covariance between MSCEIT Emotional Management and MSCEIT Emotional Relationships) fitted well: Chi^2^ (8) = 13.24, RMSEA = 0.03, CFI = 0.99, SRMR = 0.02. The Extreme Responding Scores of the six tests had loadings between 0.30 and 0.61 on the common factor. The composite reliability (McDonald’s coefficient omega) for the common factor was 0.62. In the second sample, Extreme Responding Scores of the STEU and the STEM correlated 0.64. In the third sample, the Extreme Responding Scores of the three tests correlated between 0.43 and 0.61. A confirmatory factor model with the Extreme Responding Scores of the three tests as indicators, a single factor, and additional equality restrictions on the three factor loadings, the three intercepts, and the three error variances fitted the data acceptably: Chi^2^ (6) = 21.88, RMSEA = 0.06, CFI = 0.95, SRMR = 0.06. The three standardized factor loadings were 0.70. The composite reliability (McDonald’s coefficient omega) for the common factor was 0.77. Thus, Hypothesis 2.3 that extreme responding in each of the tests of an EI test battery is substantially related and can be attributed to a single underlying factor was confirmed.

### The Nomological Networks of Principal Component Metrics

Intelligence, personality traits, self-esteem, and self-report EI were regressed on the overall Emotional Intelligence Scores, Acquiescent Responding Scores, and Extreme Responding Scores across the three samples (see Table 2).

**TABLE 2:** Nomological networks of Emotional Intelligence Scores, Acquiescent. Responding Scores, and Extreme Responding Scores.

	β_*EIS*_	β_*ARS*_	β_*ERS*_	R_*Tot*_
**Sample 1**				
Gc	0.58***	−0.07*	−0.14***	0.58***
Gv	0.45***	–0.01	−0.18***	0.46***
Gf	0.67***	–0.00	−0.21***	0.68***
Gq	0.48***	0.01	−0.16***	0.49***
Big five conscientiousness	0.21***	0.01	0.14**	0.26***
Big five extraversion	0.06	0.11**	0.14***	0.19***
Big five openness	0.31***	0.07	0.16***	0.37***
Big five agreeableness	0.36***	0.08*	0.14***	0.41***
Big five neuroticism	−0.09*	0.04	–0.02	0.11
TEIQ	0.24***	0.03	0.14***	0.30***
**Sample 2**				
Rosenberg self-esteem	0.18***	−0.18***	0.15***	0.30***
TAS: Total	−0.28***	0.22***	–0.01	0.35***
TAS: DDF	−0.16***	0.18***	0.02	0.24***
TAS: DIF	−0.22***	0.21***	–0.06	0.32***
TAS EOT	−0.26***	0.12***	0.02	0.29***
**Sample 3**				
Gc	0.35***	−0.17**	−0.18**	0.41***
Gf	0.32***	–0.07	−0.23***	0.37***
Intelligence total score	0.39***	−0.15**	−0.24***	0.45***
HEXACO Honesty/Humility	0.11*	–0.06	0.01	0.12
HEXACO Emotionality	–0.01	0.19***	–0.06	0.20**
HEXACO Extraversion	0.14**	0.03	0.14**	0.21***
HEXACO Agreeableness	–0.05	–0.05	0.09	0.11
HEXACO Conscientiousn.	0.17***	0.06	0.09	0.22***
HEXACO Openness	0.11*	–0.03	–0.05	0.12
Rosenberg Self-Esteem	0.22***	–0.02	0.10*	0.26***
TEIQue-SF	0.23***	–0.06	0.11*	0.27***
TAS: DIF	−0.18***	0.05	–0.05	0.20**
TAS: DDF	−0.28***	0.20***	–0.07	0.35***
TAS: EOT	−0.26***	0.02	–0.01	0.26***
TAS: Total	−0.27***	0.04	–0.03	0.28***

Regression analyses were executed with the variables in the nomological network as criteria and Emotional Intelligence Scores (EIS), Acquiescent Responding Scores (ARS), and Extremity Responding Scores (ERS) as predictors. R_Tot_ represents the multiple correlation of Emotional Intelligence Scores, Acquiescent Responding Scores, and Extreme Responding Scores with each of the variables in the nomological network. DIF: Difficulty Identifying Feelings, DDT: Difficulty Describing Feelings, EOT: Externally Oriented Thinking. ***p < 0.001, **p < 0.01, *p < 0.05.

#### Nomological Network of EI

As predicted by Hypothesis 3.1, EI had the strongest relationships with the intelligence tests. The relationships were medium to large. There were small, but consistent relationships with self-report EI (positive correlations with the TEIQue and negative correlations with the TAS-scales). There were small positive relationships with self-esteem. There were small to medium relations with the Big Five/Big Six personality traits. However, only conscientiousness and openness were consistently positively related to EI.

#### Nomological Network of Acquiescent Responding

There were small, but significant negative correlations of Acquiescent Responding Scores with crystalized intelligence both in sample 1 and in sample 3, but not with other forms of intelligence. No consistent relationships of Acquiescent Responding Scores with personality were found between the samples. Thus, Hypothesis 3.2 was only partially confirmed. Only some evidence was found for a negative relationship of acquiescent responding with crystalized intelligence.

#### Nomological Network of Extreme Responding

Consistent, but small negative relationships were observed between Extreme Responding Scores and all intelligence tests. A small positive relationship was observed of Extreme Responding Scores with extraversion as well as with the Rosenberg Self-Esteem scale. The relationships with the other personality traits and self-report EI were inconsistent across the samples. Thus, with respect to Hypothesis 3.3. only the negative relationship between extreme responding and intelligence was clearly confirmed.

### Relationships Between Profile Similarity Metrics and Principal Component Metrics

#### Relationships Between PSM Shape and PCM

For each of the 11 EI tests the PSM Shape was regressed on the PCM Emotional Intelligence Scores, Acquiescent Responding Scores, and Extreme Responding Scores (see Table 3). As predicted, there were strong positive relationships between PSM Shape and PCM Emotional Intelligence Scores (see Hypothesis 4.1). The standardized regression coefficients ranged from 0.68 to 0.96. Furthermore, there were either non-significant or significant, but small relationships between PSM Shape and PCM Acquiescent Responding Scores for 10 of the 11 EI tests. A moderate positive relationship was observed between PSM Shape and PCM Acquiescent Responding Scores for MSCEIT Pictures (β = 0.37). Furthermore, there were significant negative relationships between PSM Shape and PCM Extreme Responding Scores for 10 of the 11 EI tests. This relationship was moderate to large for four of the 11 EI-tests (STEM-R, STEU, STEM, GERT-R).

**TABLE 3:** Regression of PSM shape on PCM Emotional Intelligence Scores, Acquiescent Responding Scores, and Extreme responding Scores for each EI test.

EI Test	β_*EIS*_	β_*ARS*_	β_*ERS*_	*R^2^_*Tot*_*
**Sample 1: MSCEIT (*N* = 713)**				
Faces	0.87***	0.01	−0.29***	0.84
Pictures	0.79***	0.37***	0.01	0.76
Sensations	0.94***	0.07***	−0.20***	0.92
Facilitation	0.89***	−0.11***	−0.22***	0.85
Emotional Management	0.96***	0.12***	−0.17***	0.96
Emotional Relationships	0.95***	0.03**	−0.17***	0.93
**Sample 2 (*N* = 828)**				
STEU	0.68***	−0.14***	−0.64***	0.84
STEM	0.74***	–0.01	−0.57***	0.87
**Sample 3 (*N* = 762)**				
STEU-R	0.74***	−0.24***	−0.24***	0.91
STEM-R	0.80***	−0.14***	−0.42***	0.86
GERT-R	0.74***	−0.14***	−0.57***	0.87

Regression analyses were executed with the PSM Shape as criterion and Emotional Intelligence Scores (EIS), Acquiescent Responding Scores (ARS), and Extremity Responding Scores (ERS) as predictors. R^2^_Tot_ represents the proportion of variance accounted for by Emotional Intelligence Scores, Acquiescent Responding Scores, and Extreme Responding Scores for each of the EI tests. ***p < 0.001, **p < 0.01, *p < 0.05.

#### Relationships Between PSM Elevation and PCM

For each of the 11 EI tests the PSM Elevation was regressed on the PCM Emotional Intelligence Scores, Acquiescent Responding Scores, and Extreme Responding Scores (see Table 4). As predicted, strong positive relationships were observed between PSM Elevation and PCM Acquiescent Responding Scores for all 11 EI tests (ranging from 0.55 to 0.98) (see Hypothesis 4.2). Surprisingly, the relationship between PSM Elevation and PCM Emotional Intelligence Scores varied substantially (from −0.80 to 0.38). In an exploratory search to account for this variation, it was found that the mean item score across all items in an EI test played a central role. When the mean item score was low, which meant that most items were incorrect, a negative relationship was observed. Participants with a high EI ability displayed less score elevation. When the mean item score was high, which meant that most items were correct, a positive relation was observed. Participants with a high EI ability displayed on average more score elevation. The variation in the relationship between PSM Elevation and PCM Emotional Intelligence Scores correlated 0.95 with the mean item score across the 11 EI ability tests (see also Supplementary Figure 1). Finally, there were small, near zero relationships between PSM Elevation and PCM Extreme Responding Scores (ranging from −0.06 to 0.04).

**TABLE 4:** Regression of PSM elevation on PCM Emotional Intelligence Scores, Acquiescent Responding Scores, and Extreme Responding Scores for each EI test.

EI Test	β_*EIS*_	β_*ARS*_	β_*ERS*_	*R* ^2^_*Tot*_
**Sample 1: MSCEIT (*N* = 713)**				
Faces	−0.75***	0.66***	0.01*	0.98
Pictures	−0.80***	0.55***	−0.06***	0.98
Facilitation	−0.47***	0.83***	0.03**	0.90
Sensations	−0.08***	0.98***	–0.01	0.97
Emotional Management	0.37***	0.88***	0.00	0.90
Emotional Relationships	−0.11***	0.91***	0.00	0.84
**Sample 2 (*N* = 828)**				
STEU	0.22***	0.97***	−0.01***	0.99
STEM	0.38***	0.92***	−0.04***	0.97
**Sample 3 (*N* = 762)**				
STEU-R	−0.03***	0.98***	−0.02*	0.97
STEM-R	0.18***	0.97***	−0.02***	0.97
GERT-R	−0.34***	0.94***	0.04***	0.99

Regression analyses were executed with the PSM Elevation as criterion and PCM Emotional Intelligence Scores (EIS), Acquiescent Responding Scores (ARS), and Extremity Responding Scores (ERS) as predictors. R^2^_Tot_ represents the proportion of variance accounted for by Emotional Intelligence Scores, Acquiescent Responding Scores, and Extreme Responding Scores for each of the EI tests. ***p < 0.001, **p < 0.01, *p < 0.05.

#### Relationships Between PSM Scatter and PCM

For each of the 11 EI tests the PSM Scatter was regressed on the PCM Emotional Intelligence Scores, Acquiescent Responding Scores, and Extreme Responding Scores (see Table 5). As expected, positive relationships were observed between PSM Scatter and PCM Extreme Responding Scores ranging from 0.19 to 0.62 (see Hypothesis 4.3). In addition, the predicted positive relationships between PSM Scatter and PCM Emotional Intelligence Scores were confirmed for 10 of the 11 EI tests (ranging from 0.41 to 0.88). Only for MSCEIT Pictures an unexpected negative relation was observed (β = −0.12). Finally, a large variation in relationships between PSM Scatter and PCM Acquiescent Responding Scores was observed ranging from −0.32 to 0.91. An exploration of the variation of this relationship also revealed that the mean item score across all items played a central role. When the overall mean item score was low (which meant that most items were incorrect), acquiescent responding related to more scatter. When the overall mean item score was high (which meant that most items were correct), acquiescent responding related to less scatter. The variation in the relationship between PSM Scatter and PCM Acquiescent Responding Scores correlated −0.99 with the mean item score (see also Supplementary Figure 2).

**TABLE 5:** Regression of PSM scatter on PCM Emotional Intelligence Scores, Acquiescent Responding Scores, and Extreme Responding Scores for each EI test.

EI Test	β_*EIS*_	β_*ARS*_	β_*ERS*_	*R* ^2^_*Tot*_
**Sample 1: MSCEIT (*N* = 713)**				
Faces	0.41***	0.91***	0.47***	0.76
Pictures	−0.12***	0.78***	0.19***	0.52
Facilitation	0.69***	0.32***	0.62***	0.88
Sensations	0.78***	0.22***	0.48***	0.88
Emotional Management	0.76***	−0.11***	0.57***	0.90
Emotional Relationships	0.77***	0.03*	0.55***	0.89
**Sample 2 (*N* = 828)**				
STEU	0.73***	−0.29***	0.50***	0.93
STEM	0.72***	−0.32***	0.58***	0.91
**Sample 3 (*N* = 762)**				
STEU-R	0.79***	−0.23***	0.47***	0.91
STEM-R	0.75***	−0.23***	0.49***	0.82
GERT-R	0.88***	0.01	0.38***	0.91

Regression analyses were executed with the PSM Scatter as criterion and PCM Emotional Intelligence Scores (EIS), Acquiescent Responding Scores (ARS), and Extremity Responding Scores (ERS) as predictors. R^2^_Tot_ represents the proportion of variance accounted for by Emotional Intelligence Scores, Acquiescent Responding Scores, and Extreme Responding Scores for each of the EI tests. ***p < 0.001, **p < 0.01, *p < 0.05.

## Discussion

### EI Abilities

The central hypothesis (Hypothesis 1.1) was fully confirmed in all samples and for all 11 EI tests. A bipolar component emerged in all EI tests and reliable component scores could be computed. Moreover, the interpretation of these 11 bipolar components as EI abilities was supported by their internal structure across EI tests in a test battery and the nomological network of the overall EI score. The bipolar components of different EI tests were substantially correlated and fitted a unifactorial model using confirmatory factor analysis (Hypothesis 2.1). Moreover, the overall EI score derived from the bipolar EI components correlated highest with measurements of classical intelligence, and demonstrated near zero, small, or moderate relationships with measurements of self-reported EI, personality, well-being, and psychopathology (Hypothesis 3.1). Thus, the Principal Component Metrics are the first to demonstrate that there is one EI ability per (sub)test that accounts for the item scores (next to response tendencies and item specific personality factors) for 11 (9) EI (sub)tests.

The prediction with respect to the precise pattern of the item loadings was also systematically confirmed. Based on the observation of a high correlation between lay and expert evaluations of correctness (Mayer et al., 2003), it was hypothesized that the loadings on the bipolar EI component would mirror the mean item scores (see Hypothesis 1.1). For all 11 EI tests the congruence between the predicted and observed component loadings (Tucker’s phi) was very high (0.96 and higher). This finding empirically supports the use of consensus to identify the correctness of items in the emotional intelligence domain. The judgment of the majority about (in)correctness of EI items is reflected in the correlational and component structure of EI items.

From an assessment perspective, this finding implies that there are strong relationships between the difficulty and the validity of EI items: The easier the EI item (the more participants score the item correctly), the more valid the item is as an indicator of the EI construct (the higher its absolute loading on the EI component). With classic ability items there is no relationship between the difficulty of an item (how many respondents get the item correct) and the discrimination of an item (how well the item differentiates those who are low or high on the ability). This means that should one apply a Likert rating scale format to classic ability items, one would still expect a bipolar ability factor with correct items loading positively and incorrect items loading negatively. However, for these classic ability items no relationships of the average item scores with item correctness and with item loadings on the bipolar ability factor would be expected. For instance, classic multiple-choice items work with attractive distractors that are wrong. These distractors would get a high average score when rated on a Likert response scale. However, as they are wrong, they would load negatively on the ability factor. We suggest two characteristics that differentiate (cognition about) the emotion domain from other cognitive domains that can possibly account for this finding. First, all people are constantly exposed to their own and others’ emotional processes, while there is large interindividual variability in the exposure to other cognitive domains (such as exposure to math or exposure to difficult vocabulary). Second, emotion processes are natural processes that are probabilistically organized. They are not fixed processes elicited by necessary and sufficient conditions. From a theoretical perspective there is a large consensus that emotions have to be conceptualized as multi-componential processes that are elicited by goal-relevant experiences (e.g., Scherer, 2005). The commonly recognized components are the appraisal component, the action tendency component, the bodily reaction component, the expression component, and the feeling component. The relationships between these components are probabilistic: some relationships are more likely than others. It has even been demonstrated that the multicomponential nature of emotions and the probabilistic relationships between the components is universally encoded in language (e.g., Fontaine et al., 2013). As all people are constantly exposed to their own and others emotional processes, there are abundant opportunities to learn from experience for everybody. Thus, those emotional processes that have a high probability to occur and point with a high probability to the presence or absence of a specific emotion or emotion characteristic, will also be learned and recognized as such by most people. Information that does not differentiate well between the presence or absence of an emotion process or an emotion characteristic, will not be learned easily. However, that information is not well suited to discriminate people with high and low abilities in the emotion domain. Good reasons can be given for why it will sometimes be compatible with the presence and sometimes be compatible with the absence of a specific emotion process or emotion characteristic.

### Response Tendencies

In all three samples and for all 11 EI tests an acquiescent responding component was identified, with a clear tendency for items with a mean score close to the mean of the response scale to load higher on this component (see Hypothesis 1.2). There is, however, a large variation in the internal consistency of the acquiescent responding component scores across each (sub)test. This variation can be accounted for by differences in the designs of the EI tests. In the MSCEIT tests there are only a few items with a mean score close to the response scale mean, while in the STEU and the STEM there are many such items. The more of these items in a test, more reliable the Acquiescent Responding Scores are as these items define the acquiescent responding component the best.

The typically used indicator for extreme responding, the proportion of extreme responses across items, was – as predicted – highly correlated with EI ability (see Hypothesis 1.3). Respondents that are high in EI are more likely to select the extreme scale scores for items that are either clearly incorrect or clearly correct. This finding is at odds with the assumption of Legree et al. (2014) that the scatter of the responses across EI items is irrelevant for assessing EI abilities. Based on the current findings, it can be concluded that scatter confounds extreme responding with valid individual differences in EI ability.

Depending on the design of the instrument the proportion of extreme responses is also related to acquiescent responding. The varying relationships with acquiescent responding can be accounted for by the design of the tests. When most items are incorrect, acquiescent responding leads to a lower proportion of extreme responses and when most items are correct, acquiescent responding leads to a higher proportion of extreme responses. Thus, to assess extreme responding, the proportion of extreme responses should be statistically controlled for both in EI ability and acquiescent responding.

There was clear evidence for the stability of extreme responding across EI tests. There were substantial relationships between extreme responding scores across EI tests, and the extreme responding scores fitted a unifactorial structure using confirmatory factor analysis (see Hypothesis 2.3). Of the expected nomological network only small, but consistent negative relationship with intelligence were observed (see Hypothesis 3.3). People who score higher on classical intelligence tests show less extreme responding. A possible explanation for this finding is that more intelligent people are not only more likely to know the correct answer, but are also more likely to know when they do not know or are uncertain about the correct answer. When uncertain about the correct answer, one is less likely to make a large mistake when using midpoint scale scores. Thus, while participants are asked to rate the correctness (or effectiveness) of EI items, the confidence they have in their answers is also likely to play a role in how they answer.

Compared to acquiescent responding, more evidence is found for stable interindividual differences in extreme responding (with a more reliable extreme responding factor using CFA and more consistent negative relationships with intelligence). Possibly this is not only to be accounted for by the design of EI tests (with the MSCEIT tests having unreliable acquiescent responding scores), but also because Likert response scales are applied in the context of maximum performance assessment. In that context the more incorrect an item is, the less correct it is. This mutual exclusiveness is less the case in a typical performance context. For instance, people can pursue conflicting values in their lives, but it is unlikely that people will claim that a solution is incorrect and correct at the same time. Thus, compared to the typical performance context a maximum performance context is possibly more sensitive to individual differences in extreme responding than to individual differences in acquiescent responding. The shift from the typical to the maximum performance context could also account for the observation that the expected nomological networks of the response tendencies are not well confirmed. Being agreeable and pleasing to others is much less relevant in a maximum than a typical performance context.

### General Personality Characteristics and Specific Preferences

It was deemed *a priori* very likely that general personality traits or specific preferences would affect specific items in EI tests. However, it was also predicted that in well-designed EI tests only the EI ability and acquiescent responding should be the major constructs that account for the responses across items. In all 11 EI tests two components were sufficient to identify the expected bipolar EI component. Moreover, in 8 of the 11 tests two components were sufficient to identify the expected acquiescent responding component. Only for three EI tests an additional component was needed for the predicted bipolar EI and unipolar acquiescent responding to emerge well. For two of the three EI tests, this component represented a tendency to rate positively or negatively valenced emotion terms highly, independent of their correctness. Scores on these two additional components, however, were not correlated between the two MSCEIT tests, which points to the (sub)test specificity of these components. The third additional component did not have a general interpretation, rather it refers to interindividual differences in the preferences for specific reactions in one specific scenario. It might be noted that this was observed with the shortest EI test of the 11 tests (only 9 items and three item stems). Probably the shorter the instrument, the more specific preferences become salient in the response structure. It can be concluded that, as EI ability was identified in all 11 (sub)tests and an extra component was only needed in three of the 11 (sub)tests to identify acquiescent responding, there is very little evidence that general personality characteristics or specific preferences should be taken into account when analyzing responses to EI items.

### Relationships Between Profile Similarity Metrics and Principal Component Metrics

As predicted, there were strong relationships between PSM Shape and PCM Emotion Intelligence Scores (see Hypothesis 4.1), between PSM Elevation and PCM Acquiescent Responding Scores (see Hypothesis 4.2), and between PSM Scatter and PCM Extreme Responding Scores (see Hypothesis 4.3). These relationships fit the *a priori* model that there are three main constructs that determine the raw item scores to EI ability items: (1) Emotional Intelligence (both leading to a bipolar EI factor and affecting similarities in shape), (2) Acquiescent Responding (both leading to a unipolar EI factor and affecting score elevation across all items) and (3) Extreme Responding (both leading to an increased use of extreme response categories and affecting score scatter across all items). Moreover, the predicted relationships between PSM Scatter and PCM Emotion Intelligence Scores were by and large confirmed (see Hypothesis 4.3). Thus, score scatter does not only contain information about extreme responding but contains also valid information about maximum emotional intelligence.

Most importantly, however, not only were the predicted relationships confirmed, also many relationships emerged about which no predictions were made. First, for 10 of the 11 subtests, PSM Shape and PCM Extreme Responding Scores were negatively correlated. It is thus observed that the tendency to use more extreme responses suppresses similarity in shape. This finding cannot be explained by the fact that participants who use more extreme responses would be less emotionally intelligent. PCM Extreme Responding Scores were computed controlling for PCM Emotion Intelligence Scores and PCM Acquiescent Responding Scores. Therefore, these results seem to indicate that profile similarities are both sensitive to EI ability and a tendency for extreme responding.

Second, two phenomena were observed that were highly related: large variation was observed in the relationships (1) of PSM Elevation with PCM Emotion Intelligence Scores and (2) of PSM Scatter with PCM Acquiescent Responding Scores. The variation in these relationships could be very well accounted for by mean item score across all items in a test. When the mean item score is low – and thus most items are clearly incorrect – (1) emotional intelligence is negatively correlated with PSM Elevation (the higher the EI ability the lower the incorrect items are scored) and (2) PCM Acquiescent Responding Scores are positively correlated with PSM Scatter (people who have a higher tendency to acquiesce will show more scatter in their responses). When the mean item score is high – and thus most items are clearly correct – the opposite pattern is observed: (1) Emotional intelligence is positively correlated with PSM Elevation (the higher the EI ability score the higher the correct items are scored) and (2) PCM Acquiescent Responding Scores are negatively correlated with PSM Scatter (people who have a higher tendency to acquiesce will show less scatter in their responses).

These findings suggest that what the three Profile Similarity Metrics are measuring interacts with the design of an EI instrument. Two characteristics seem to play a central role. The first characteristic is the extent to which the EI instrument is balanced in terms of correct and incorrect items. The more an instrument is balanced, the less score elevation is related to emotional intelligence and the less score scatter is related to acquiescent responding. The second characteristic is the number of items that are neither clearly correct, nor clearly incorrect. The more such items, the more extreme responding suppresses profile similarities.

It is thus clear that when Likert response scales are used both acquiescent and extreme responding affect responses to EI ability items and their overall impact depends on the design of the EI ability instrument. This observation pleads for the construction of balanced instruments with a gradual change in the incorrectness-correctness of the items. In an instrument where there are as many correct as incorrect items the effect of acquiescence cancels out (e.g., Ten Berge, 1999). Moreover when there is a gradual change in the incorrectness-correctness of the items also the effects of extreme responding cancels out.

### Limitations and Future Direction

A limitation of the current study is that, while clear nomological networks were observed for the Emotional Intelligence Scores, few relationships were observed for the two response tendencies. The precise psychological meaning of response tendencies in the context of ability measurement and how they differ from response tendencies in the context personality assessment remain to be further explored.

While in general the results demonstrate that the Principal Component Metrics succeed in disentangling valid information about EI ability from interindividual differences in responding tendencies, even for EI tests that have unbalanced designs, the question remains to what extent this holds true for extremely unbalanced EI tests. Particularly with the MSCEIT Pictures subtest some relationships of the PCM’s deviated from the overall pattern of relationships. The specific nature of the stimuli in this subtest (abstract figures) might be responsible for these deviations, however, the highly unbalanced nature of this subtest forms an alternative explanation. This subtest is not only characterized by a very low overall item mean, but also by the complete absence of correct items. It remains to be further demonstrated that also for these extremely skewed instruments PCM still succeeds in properly disentangling EI ability from response tendencies.

Given the study’s findings it seems plausible that the Principal Component Metrics can contribute to scoring situational judgment tests outside the EI domain and provide further validity evidence. Situational judgments are often used in the industrial and organizational domain and have been demonstrated to have acceptable test-criterion relations (e.g., Christian et al., 2010). Without a clear view on the constructs that account for the reactions to situational judgment tests, the construct validity of these tests is often debated, however. An important question is whether these tests measure unidimensional constructs, or a set of different constructs that improve performance in specific contexts. Thus, unlike in the emotional intelligence domain, where it is assumed that each EI test assesses a specific EI ability, the situational judgment methodology is often applied without an assumption of unidimensionality. With the Principal Component Metrics approach highlighted throughout the current manuscript it is possible to investigate whether one or more components are needed next to acquiescent responding to account for the major sources of variation in situational judgment tests. Moreover, these different dimensions can then be easily scored using the Principal Component Metrics.

## Conclusion

The current study adds a central piece of empirical validity evidence to the EI construct: EI tests show a theoretically predicted internal structure at item level. This structure was observed on the raw item scores without the need for any transformation (as in consensus scoring). Its validity was further confirmed by the relationships between EI components across EI tests and by their nomological network. Moreover, it was demonstrated that a very simple scoring method only using information from the raw item scores can be used. The current findings thus contribute to the validity of the EI construct and the instruments that are currently used for its assessment and open new perspectives for both analyzing and scoring existing EI tests and constructing new EI tests.

## Data Availability Statement

The raw data supporting the conclusions of this article will be made available by the authors, without undue reservation.

## Ethics Statement

Sample 1 consists of a reanalysis of data reported by MacCann et al. (2014) and was collected in the United States. It satisfied the local ethical requirements. Samples 2 and 3 were collected in Belgium. Ethical review and approval was not required for the study on human participants in accordance with the local legislation and institutional requirements. The data were collected on the basis of the ethical guidelines of the Faculty of Psychology and Educational Sciences of Ghent University. Written informed consent for participation was not provided by the participants’ legal guardians/next of kin. In Belgium one is a minor till 17 years. Only participants of 18 years and older were targeted for anonymous participation in a web-based research in sample 2 and 3. Only in sample 2 one participant indicated an age of 16. Because participation was anonymous the participant could not be traced. For data integrity it was decided to keep the participant in the dataset.

## Author Contributions

JF developed the principal component metrics, executed the analyses and took the lead in writing the manuscript. ES contributed to the literature and the hypotheses development about the nomological network of the response styles. EV contributed to the empirical research of samples 2 and 3. KS contributed in adapting the Geneva Emotion Recognition Test (GERT). CM and RR shared the data of sample 1 for reanalysis and shared their expertise with respect to the MSCEIT. KRS contributed to the conceptual interpretation within an emotion framework. All authors were involved in the final interpretation of the results and finetuning of the manuscript.

## Conflict of Interest

RR was employed by RAD Science Solution. The remaining authors declare that the research was conducted in the absence of any commercial or financial relationships that could be construed as a potential conflict of interest.

## Publisher’s Note

All claims expressed in this article are solely those of the authors and do not necessarily represent those of their affiliated organizations, or those of the publisher, the editors and the reviewers. Any product that may be evaluated in this article, or claim that may be made by its manufacturer, is not guaranteed or endorsed by the publisher.
