# Supplementary material for: Assessing Emotional Intelligence Abilities, Acquiescent and Extreme Responding in Situational Judgment Tests Using Principal Component Metrics
Source: Front Psychol. 2022 Apr 26;13:813540. doi: 10.3389/fpsyg.2022.813540 (PMC9087725; doi:10.3389/fpsyg.2022.813540)
Supplement: Supplementary file 1 [file Data_Sheet_1.pdf]

Supplemental Material

Table 1

Mean Item Scores, Hypothetical Loadings, and Observed Loadings after Orthogonal Procrustes Rotation of MSCEIT Faces Test in a Two-Componential Structure

Item	Mean	H_{EI}	H_{AR}	EI	AR
Face 1_1	2.13	-.44	.41	-.27	.16
Face 1_2	2.14	-.43	.41	-.26	.61
Face 1_3	1.56	-.72	.24	-.53	.36
Face 1_4	2.50	-.25	.47	-.20	.47
Face 1_5	1.54	-.73	.23	-.55	.19
Face 2_1	1.36	-.82	.16	-.60	.15
Face 2_2	3.17	.08	.50	.17	.61
Face 2_3	2.11	-.45	.40	-.27	.72
Face 2_4	1.54	-.73	.23	-.61	.38
Face 2_5	1.32	-.84	.15	-.66	.18
Face 3_1	1.21	-.90	.10	-.70	.06
Face 3_2	4.15	.58	.33	.39	.41
Face 3_3	2.47	-.26	.46	-.17	.64
Face 3_4	1.50	-.75	.22	-.59	.29
Face 3_5	1.27	-.87	.13	-.69	.07
Face 4_1	4.17	.59	.33	.20	.40
Face 4_2	1.30	-.85	.14	-.71	-.05
Face 4_3	1.30	-.85	.14	-.74	-.04
Face 4_4	1.25	-.88	.12	-.74	-.07
Face 4_5	1.27	-.87	.13	-.74	-.09

Note. Mean: Mean item scores, H_{EI}: hypothetical loadings on Emotional Intelligence Component, H_{AR}: hypothetical loadings on Acquiescent Responding Component, EI: observed loadings on Emotional Intelligence Component, AR: observed loadings on Acquiescent Responding Component

Table 2
Mean Item Scores, Hypothetical Loadings, and Observed Loadings after Orthogonal Procrustes Rotation of MSCEIT Pictures Test in a Three-Componential Structure

Item	Mean	H_{EI}	H_{AR}	EI	AR	C3
Picture 1_1	3.65	.32	.45	-.06	.32	.26
Picture 1_2	1.44	-.78	.20	-.57	.09	.21
Picture 1_3	1.62	-.69	.26	-.65	.14	.25
Picture 1_4	1.31	-.85	.14	-.79	.03	.24
Picture 1_5	1.33	-.84	.15	-.78	.02	.22
Picture 2_1	2.74	-.13	.49	-.17	.54	.02
Picture 2_2	1.78	-.61	.31	-.59	.35	-.06
Picture 2_3	1.68	-.66	.28	-.69	.08	.10
Picture 2_4	1.98	-.51	.37	-.42	.41	-.07
Picture 2_5	1.53	-.74	.23	-.57	.13	.17
Picture 3_1	2.68	-.16	.49	-.15	.57	.19
Picture 3_2	1.40	-.80	.18	-.73	.21	-.09
Picture 3_3	1.37	-.82	.17	-.76	.15	-.12
Picture 3_4	1.70	-.65	.29	-.60	.30	.03
Picture 3_5	1.34	-.83	.16	-.74	.12	-.08
Picture 4_1	1.48	-.76	.21	-.56	.27	-.18
Picture 4_2	2.22	-.39	.42	-.21	.61	-.34
Picture 4_3	2.75	-.13	.49	-.07	.61	-.35
Picture 4_4	2.43	-.28	.46	-.24	.48	-.16
Picture 4_5	1.73	-.64	.30	-.43	.26	-.41
Picture 5_1	3.08	.04	.50	.01	.67	.34
Picture 5_2	1.38	-.81	.17	-.67	.12	-.29
Picture 5_3	1.62	-.69	.26	-.55	.26	-.44
Picture 5_4	1.59	-.71	.25	-.50	.24	-.48
Picture 5_5	1.59	-.71	.25	-.52	.01	-.56
Picture 6_1	1.74	-.63	.30	-.44	.20	.09
Picture 6_2	2.62	-.19	.48	-.03	.62	-.27
Picture 6_3	1.60	-.70	.26	-.48	.30	-.34
Picture 6_4	1.57	-.72	.24	-.52	.14	-.14
Picture 6_5	1.69	-.66	.29	-.42	.28	-.34

Note. Mean: Mean item scores, H_{EI}: hypothetical loadings on Emotional Intelligence Component, H_{AR}: hypothetical loadings on Acquiescent Responding Component, EI: observed loadings on Emotional Intelligence Component, AR: observed loadings on Acquiescent Responding Component, C3: loadings on third valence component.

Table 3

Mean Item Scores, Hypothetical Loadings, and Observed Loadings after Orthogonal Procrustes Rotation of MSCEIT Facilitation Test in a Three-Componential Structure

Item	Mean	H_{EI}	H_{AR}	EI	AR	C3
Item 1_1	1.23	-.89	.11	-.63	.04	-.01
Item 1_2	1.36	-.82	.16	-.57	.13	.15
Item 1_3	4.82	.91	.09	.56	.21	.03
Item 2_1	2.20	-.40	.42	-.48	.17	.22
Item 2_2	4.30	.65	.29	.54	.40	.16
Item 2_3	1.70	-.65	.29	-.66	.06	.04
Item 3_1	1.92	-.54	.35	-.31	.25	-.52
Item 3_2	1.33	-.84	.15	-.59	.22	-.30
Item 3_3	3.99	.50	.38	.41	.23	.37
Item 4_1	2.37	-.31	.45	-.16	.51	.49
Item 4_2	2.65	-.18	.48	-.13	.58	-.47
Item 4_3	2.43	-.28	.46	-.12	.39	-.61
Item 5_1	2.23	-.39	.43	-.19	.54	.33
Item 5_2	3.99	.50	.38	.47	.08	.24
Item 5_3	1.64	-.68	.27	-.53	.14	-.37

Note. Mean: Mean item scores, H_{EI}: hypothetical loadings Emotional Intelligence Component, H_{AR}: hypothetical loadings on Acquiescent Responding Component, EI: observed loadings on Emotional Intelligence Component, AR: observed loadings on Acquiescent Responding Component, C3: loadings on third valence component.

Table 4

Mean Item Scores, Hypothetical Loadings, and Observed Loadings after Orthogonal Procrustes Rotation of MSCEIT Sensations Test in a Two-Componential Structure

Item	Mean	H_{EI}	H_{AR}	EI	AR
Item 1_1	3.44	.22	.48	.26	.61
Item 1_2	3.47	.24	.47	.26	.56
Item 1_3	1.45	-.78	.20	-.63	.16
Item 2_1	4.20	.60	.32	.67	.33
Item 2_2	2.59	-.21	.48	-.04	.45
Item 2_3	1.71	-.65	.29	-.63	.14
Item 3_1	3.19	.09	.50	.16	.46
Item 3_2	3.65	.32	.45	.41	.46
Item 3_3	2.10	-.45	.40	-.42	.42
Item 4_1	4.00	.50	.38	.55	.33
Item 4_2	2.23	-.39	.43	-.35	.33
Item 4_3	1.91	-.55	.35	-.57	.28
Item 5_1	4.27	.63	.30	.72	.21
Item 5_2	1.73	-.64	.30	-.62	.24
Item 5_3	2.22	-.39	.42	-.44	.25

Note. Mean: Mean item scores, H_{EI}: hypothetical loadings on Emotional Intelligence Component, H_{AR}: hypothetical loadings on Acquiescent Responding Component, EI: observed loadings on Emotional Intelligence Component, AR: observed loadings on Acquiescent Responding Component.

Table 5

Mean Item Scores, Hypothetical Loadings, and Observed Loadings after Orthogonal Procrustes Rotation of MSCEIT Emotional Management Test a Two-Componential Structure

Item	Mean	H_{EI}	H_{AR}	EI	AR
Item 1_1	4.07	.54	.36	.65	.19
Item 1_2	4.18	.59	.33	.61	.16
Item 1_3	1.87	-.57	.34	-.59	.10
Item 1_4	3.68	.34	.44	.28	.47
Item 2_1	3.77	.39	.43	.53	.19
Item 2_2	2.49	-.25	.47	-.34	.27
Item 2_3	3.59	.29	.46	.49	.09
Item 2_4	1.97	-.52	.37	-.60	.23
Item 3_1	4.09	.54	.35	.54	.30
Item 3_2	3.94	.47	.39	.49	.43
Item 3_3	4.21	.61	.32	.62	.35
Item 3_4	3.85	.43	.41	.35	.48
Item 4_1	4.08	.54	.35	.57	.29
Item 4_2	4.30	.65	.29	.73	.27
Item 4_3	2.32	-.34	.44	-.38	.34
Item 4_4	2.39	-.30	.45	-.40	.49
Item 5_1	1.76	-.62	.31	-.67	.06
Item 5_2	3.83	.42	.41	.52	.13
Item 5_3	2.01	-.50	.38	-.51	.22
Item 5_4	1.82	-.59	.33	-.46	.15

Note. Mean: Mean item scores, H_{EI}: hypothetical loadings on Emotional Intelligence Component, H_{AR}: hypothetical loadings on Acquiescent Responding Component, EI: observed loadings on Emotional Intelligence Component, AR: observed loadings on Acquiescent Responding Component.

Table 6

Mean Item Scores, Hypothetical Loadings, and Observed Loadings after Orthogonal Procrustes Rotation of MSCEIT Emotional Relationships Test a Three-Componential Structure

Item	Mean	H_{EI}	H_{AR}	EI	AR	C3
Item 1_1	4.05	.73	.02	.73	.09	-.08
Item 1_2	2.00	-.63	.19	-.62	.44	.00
Item 1_3	1.80	-.74	-.13	-.74	.12	-.17
Item 2_1	4.02	.71	.30	.72	.26	.15
Item 2_2	1.91	-.62	.07	-.62	.31	-.06
Item 2_3	2.15	-.64	-.15	-.65	.14	-.21
Item 3_1	3.61	.54	-.10	.53	.44	-.40
Item 3_2	2.53	-.26	.91	-.21	.48	.79
Item 3_3	4.14	.51	-.10	.49	.35	-.36

Note. Mean: Mean item scores, H_{EI}: hypothetical loadings on Emotional Intelligence Component, H_{AR}: hypothetical loadings on Acquiescent Responding Component, EI: observed loadings on Emotional Intelligence Component, AR: observed loadings on Acquiescent Responding Component, C3: loadings on third scenario-specific component.

Table 7
Mean Item Scores, Hypothetical Loadings, and Observed Loadings after Orthogonal Procrustes Rotation of STEU in a Two-Componential Structure

Item	Mean	H_{EI}	H_{AR}	EI	AR
STEU1_2	3.26	-.43	.41	-.10	.38
STEU1_3	3.17	.13	.49	.19	.33
STEU1_4	4.01	.09	.50	.16	.28
STEU1_5	3.68	.51	.37	.31	.15
STEU2_1	2.74	.34	.44	.22	.21
STEU2_2	4.71	-.13	.49	-.09	.46
STEU2_3	4.45	.86	.13	.52	.10
STEU2_4	3.14	.73	.24	.37	.27
STEU2_5	4.54	.07	.50	-.10	.49
STEU3_1	1.47	.77	.20	.47	.13
STEU3_2	3.20	-.77	.21	-.37	.14
STEU3_3	4.61	.10	.50	-.01	.32
STEU3_4	1.41	.80	.18	.47	.03
STEU3_5	4.36	-.80	.18	-.38	.13
STEU4_1	2.53	.68	.27	.42	.12
STEU4_2	4.73	-.24	.47	-.16	.52
STEU4_3	4.01	.87	.12	.50	.11
STEU4_4	1.33	.51	.37	.22	.37
STEU4_5	3.44	-.84	.15	-.36	.13
STEU5_1	2.53	.22	.48	.05	.37
STEU5_2	4.36	-.23	.47	-.09	.47
STEU5_3	3.62	.68	.27	.30	.21
STEU5_4	4.07	.31	.45	.08	.37
STEU5_5	3.24	.54	.36	.28	.29
STEU6_1	4.49	.12	.49	.09	.42
STEU6_2	4.39	.74	.22	.44	.10
STEU6_3	3.48	.70	.26	.40	.23
STEU6_4	2.74	.24	.47	.04	.46
STEU6_5	2.53	-.13	.49	-.17	.55
STEU7_1	3.12	-.24	.47	-.15	.51
STEU7_2	4.03	.06	.50	-.02	.42
STEU7_3	4.06	.51	.37	.32	.28
STEU7_4	3.50	.53	.36	.37	.30
STEU7_5	3.47	.25	.47	.10	.44
STEU8_1	1.99	.24	.47	.19	.31
STEU8_2	4.49	-.51	.37	-.28	.33
STEU8_3	3.49	.75	.22	.42	.08
STEU8_4	4.04	.25	.47	.10	.41
STEU8_5	1.88	.52	.36	.23	.29
STEU9_1	4.30	-.56	.34	-.28	.34
STEU9_2	2.81	.65	.29	.29	.18
STEU9_3	3.46	-.09	.50	-.07	.41
STEU9_4	3.14	.23	.47	.14	.34
STEU9_5	4.02	.07	.50	-.02	.36
STEU10_1	4.42	.51	.37	.41	.16
STEU10_2	3.72	.71	.25	.42	.13

STEU10_3	3.98	.36	.44	.11	.36
STEU10_4	3.05	.49	.38	.22	.30
STEU10_5	2.88	.03	.50	-.10	.43
STEU11_1	3.88	-.06	.50	-.12	.45
STEU11_2	4.18	.44	.40	.28	.29
STEU11_3	3.12	.59	.33	.43	.15
STEU11_4	1.61	.06	.50	.00	.37
STEU11_5	3.54	-.70	.26	-.51	.32
STEU12_1	1.90	.27	.46	.24	.43
STEU12_2	3.75	-.55	.35	-.22	.41
STEU12_3	3.87	.38	.43	.24	.30
STEU12_4	2.41	.44	.40	.32	.20
STEU12_5	2.88	-.30	.46	-.13	.46
STEU13_1	3.65	-.06	.50	.06	.45
STEU13_2	3.79	.33	.45	.18	.36
STEU13_3	3.38	.39	.42	.28	.46
STEU13_4	3.67	.19	.48	.16	.39
STEU13_5	2.04	.34	.44	.20	.45
STEU14_1	3.99	-.48	.38	-.26	.19
STEU14_2	4.27	.49	.38	.27	.33
STEU14_3	2.05	.64	.30	.51	.22
STEU14_4	2.35	-.47	.39	-.39	.42
STEU14_5	3.08	-.32	.45	-.15	.39
STEU15_1	2.78	.04	.50	.03	.49
STEU15_2	4.08	-.11	.49	-.07	.35
STEU15_3	4.21	.54	.35	.26	.28
STEU15_4	3.72	.60	.32	.47	.11
STEU15_5	2.88	.36	.43	.25	.30
STEU16_1	2.55	-.06	.50	-.14	.45
STEU16_2	3.93	-.22	.48	-.09	.40
STEU16_3	3.35	.46	.39	.31	.23
STEU16_4	2.67	.18	.48	.11	.36
STEU16_5	2.05	-.17	.49	-.04	.42
STEU17_1	1.54	-.47	.39	-.23	.35
STEU17_2	1.55	-.73	.23	-.43	.28
STEU17_3	3.55	-.73	.24	-.40	.30
STEU17_4	4.55	.28	.46	.10	.46
STEU17_5	4.21	.77	.20	.52	.11
STEU18_1	2.21	.60	.32	.27	.37
STEU18_2	3.11	-.40	.42	-.16	.48
STEU18_3	3.93	.06	.50	.00	.44
STEU18_4	4.21	.47	.39	.29	.40
STEU18_5	3.53	.61	.32	.44	.14
STEU19_1	3.52	.27	.46	.22	.40
STEU19_2	1.94	.26	.47	.05	.32
STEU19_3	3.44	-.53	.36	-.35	.34
STEU19_4	4.39	.22	.48	.14	.28
STEU19_5	2.08	.70	.26	.45	-.08
STEU20_1	2.11	-.46	.40	-.33	.38
STEU20_2	2.55	-.45	.40	-.26	.36

STEU20_3	4.08	-.23	.47	-.13	.42
STEU20_4	2.72	.54	.35	.33	.01
STEU20_5	3.37	-.14	.49	-.06	.44
STEU21_1	3.98	.18	.48	.08	.28
STEU21_2	3.21	.49	.38	.30	.32
STEU21_3	4.13	.11	.49	.08	.44
STEU21_4	3.21	.56	.34	.39	.36
STEU21_5	3.84	.10	.49	.01	.42
STEU22_1	3.27	.42	.41	.32	.37
STEU22_2	3.05	.13	.49	-.02	.50
STEU22_3	4.51	.03	.50	-.04	.47
STEU22_4	4.14	.76	.21	.49	.02
STEU22_5	3.15	.57	.34	.38	.31
STEU23_1	4.41	.08	.50	.05	.54
STEU23_2	3.53	.70	.25	.41	.25
STEU23_3	4.57	.27	.46	.13	.56
STEU23_4	3.58	.78	.19	.60	.20
STEU23_5	1.43	.29	.46	.06	.54
STEU24_1	4.63	-.78	.19	-.54	.22
STEU24_2	3.61	.81	.17	.56	.23
STEU24_3	4.06	.31	.45	.05	.51
STEU24_4	4.76	.53	.36	.26	.47
STEU24_5	2.81	.88	.11	.56	.09
STEU25_1	1.77	-.09	.50	-.02	.52
STEU25_2	3.49	-.61	.31	-.42	.38
STEU25_3	3.99	.24	.47	.22	.29
STEU25_4	1.84	.49	.38	.40	.01
STEU25_5	4.36	-.58	.33	-.43	.37
STEU26_1	3.22	.68	.27	.44	-.16
STEU26_2	3.60	.11	.49	.11	.45
STEU26_3	3.47	.30	.46	.15	.38
STEU26_4	1.48	.23	.47	.07	.30
STEU26_5	4.37	-.76	.21	-.57	.26
STEU27_1	2.61	.68	.27	.44	.17
STEU27_2	3.89	-.19	.48	-.06	.42
STEU27_3	2.52	.45	.40	.30	.13
STEU27_4	1.48	-.24	.47	-.15	.36
STEU27_5	2.87	-.76	.21	-.47	.28
STEU28_1	4.75	-.07	.50	.04	.45
STEU28_2	4.57	.87	.12	.51	.20
STEU28_3	3.37	.79	.19	.45	.27
STEU28_4	4.63	.18	.48	.01	.51
STEU28_5	1.97	.82	.17	.43	.30
STEU29_1	2.58	-.51	.37	-.23	.10
STEU29_2	3.51	-.21	.48	-.05	.24
STEU29_3	3.55	.26	.47	.16	.26
STEU29_4	3.93	.27	.46	.08	.33
STEU29_5	2.46	.46	.39	.28	.09
STEU30_1	3.80	-.27	.46	-.14	.37
STEU30_2	3.61	.40	.42	.25	.41

STEU30_3	3.82	.30	.45	.16	.52
STEU30_4	4.16	.41	.42	.32	.47
STEU30_5	1.95	.58	.33	.43	.19
STEU31_1	4.00	-.53	.36	-.30	.10
STEU31_2	4.14	.50	.37	.23	.32
STEU31_3	4.55	.57	.34	.34	.41
STEU31_4	3.60	.77	.20	.59	.13
STEU31_5	1.55	.30	.45	.09	.49
STEU32_1	1.53	-.73	.24	-.43	.27
STEU32_2	3.88	-.74	.23	-.51	.33
STEU32_3	4.01	.44	.40	.28	.28
STEU32_4	4.36	.51	.37	.25	.23
STEU32_5	1.72	.68	.27	.52	.10
STEU33_1	4.11	-.64	.30	-.37	.31
STEU33_2	3.61	.55	.35	.37	.06
STEU33_3	1.75	.30	.45	.17	.31
STEU33_4	4.31	-.63	.30	-.43	.22
STEU33_5	1.64	.65	.29	.41	-.02
STEU34_1	1.89	-.68	.27	-.42	.24
STEU34_2	4.26	-.56	.35	-.38	.42
STEU34_3	4.10	.63	.30	.43	.23
STEU34_4	2.90	.55	.35	.37	.30
STEU34_5	4.19	-.05	.50	-.02	.48
STEU35_1	2.66	.59	.32	.43	.29
STEU35_2	3.40	-.17	.49	-.07	.46
STEU35_3	3.20	.20	.48	.02	.45
STEU35_4	3.55	.10	.50	.06	.51
STEU35_5	2.18	.27	.46	.16	.37
STEU36_1	4.03	-.41	.42	-.28	.40
STEU36_2	1.41	.52	.37	.29	.32
STEU36_3	3.73	-.80	.18	-.62	.24
STEU36_4	4.18	.37	.43	.12	.23
STEU36_5	3.59	.59	.33	.44	.29
STEU37_1	3.87	.29	.46	.23	.50
STEU37_2	3.06	.43	.41	.34	.13
STEU37_3	2.71	.03	.50	.06	.39
STEU37_4	2.37	-.14	.49	-.06	.52
STEU37_5	3.86	-.31	.45	-.13	.45
STEU38_1	3.98	.43	.41	.30	.22
STEU38_2	3.81	.49	.38	.32	.07
STEU38_3	2.10	.40	.42	.30	.33
STEU38_4	3.08	-.45	.40	-.36	.48
STEU38_5	2.83	.04	.50	-.05	.39
STEU39_1	3.33	-.08	.50	-.04	.58
STEU39_2	3.70	.17	.49	.17	.28
STEU39_3	3.46	.35	.44	.28	.29
STEU39_4	2.75	.23	.47	.20	.35
STEU39_5	1.99	-.13	.49	-.11	.43
STEU40_1	3.91	-.51	.37	-.39	.44
STEU40_2	2.68	.46	.40	.19	.30

STEU40_3	3.00	-.16	.49	-.16	.59
STEU40_4	4.03	.00	.50	-.12	.57
STEU40_5	4.73	.52	.37	.25	.42
STEU41_1	3.86	.86	.13	.49	.10
STEU41_2	3.00	.43	.41	.23	.11
STEU41_3	3.45	.00	.50	-.03	.37
STEU41_4	3.41	.22	.48	.10	.29
STEU41_5	3.66	.20	.48	.08	.38
STEU42_1	3.35	.33	.45	.13	.26
STEU42_2	4.75	.17	.48	-.08	.50
STEU42_3	4.13	.88	.12	.55	.05
STEU42_4	4.72	.56	.34	.25	.50
STEU42_5	3.12	.86	.13	.54	.18

Note. Mean: Mean item scores, H_{EI}: hypothetical loadings on Emotional Intelligence Component, H_{AR}: hypothetical loadings on Acquiescent Responding Component, EI: observed loadings on Emotional Intelligence Component, AR: observed loadings on Acquiescent Responding Component.

Table 8

Mean Item Scores, Hypothetical Loadings, and Observed Loadings after Orthogonal Procrustes Rotation of STEM in a Two-Componential Structure

Item	Mean	H_{EI}	H_{AR}	EI	AR
STEM1_1	3.92	.46	.39	.20	.22
STEM1_2	2.51	-.24	.47	-.14	.31
STEM1_3	4.22	.61	.31	.42	.08
STEM1_4	2.54	-.23	.47	-.22	.29
STEM2_1	3.60	.30	.46	.09	.31
STEM2_2	4.00	.50	.38	.33	.19
STEM2_3	3.64	.32	.45	.16	.21
STEM2_4	3.88	.44	.40	.22	.12
STEM3_1	4.14	.57	.34	.46	.15
STEM3_2	2.93	-.03	.50	-.13	.24
STEM3_3	3.69	.34	.44	.16	.40
STEM3_4	3.60	.30	.46	.26	.09
STEM4_1	2.39	-.31	.45	-.27	.30
STEM4_2	4.20	.60	.32	.49	.21
STEM4_3	4.20	.60	.32	.51	.12
STEM4_4	4.10	.55	.35	.45	.18
STEM5_1	3.79	.39	.42	.32	.22
STEM5_2	4.10	.55	.35	.48	.08
STEM5_3	1.99	-.50	.37	-.37	.30
STEM5_4	2.24	-.38	.43	-.19	.13
STEM6_1	2.80	-.10	.50	-.23	.27
STEM6_2	3.69	.35	.44	.27	.12
STEM6_3	3.16	.08	.50	.08	.19
STEM6_4	3.55	.27	.46	.19	.07
STEM7_1	3.50	.25	.47	.03	.48
STEM7_2	3.93	.46	.39	.22	.35
STEM7_3	4.28	.64	.30	.52	.09
STEM7_4	2.82	-.09	.50	-.03	.10
STEM8_1	4.07	.54	.36	.38	.29
STEM8_2	3.36	.18	.48	-.04	.46
STEM8_3	4.28	.64	.30	.51	.14
STEM8_4	4.15	.58	.33	.47	.08
STEM9_1	3.96	.48	.39	.28	.27
STEM9_2	4.29	.64	.29	.60	.06
STEM9_3	2.23	-.38	.43	-.35	.25
STEM9_4	3.23	.11	.49	.08	.23
STEM10_1	2.43	-.29	.46	-.21	.34
STEM10_2	3.58	.29	.46	.21	.11
STEM10_3	4.00	.50	.38	.32	.17
STEM10_4	3.35	.17	.49	.02	.33
STEM11_1	2.75	-.12	.49	-.22	.43
STEM11_2	3.56	.28	.46	.26	.19
STEM11_3	4.34	.67	.28	.65	.10
STEM11_4	3.63	.32	.45	.05	.32
STEM12_1	2.67	-.16	.49	-.14	.34
STEM12_2	4.30	.65	.29	.63	.14

STEM12_3	4.14	.57	.34	.52	.24
STEM12_4	2.17	-.42	.41	-.29	.21
STEM13_1	3.06	.03	.50	-.09	.33
STEM13_2	2.39	-.30	.45	-.31	.24
STEM13_3	3.92	.46	.39	.22	.20
STEM13_4	3.89	.45	.40	.24	.18
STEM14_1	3.34	.17	.49	.10	.38
STEM14_2	4.18	.59	.33	.44	.18
STEM14_3	2.01	-.50	.38	-.47	.26
STEM14_4	4.21	.60	.32	.41	.13
STEM15_1	3.76	.38	.43	.11	.41
STEM15_2	3.96	.48	.39	.29	.13
STEM15_3	3.46	.23	.47	.09	.32
STEM15_4	3.88	.44	.40	.23	.22
STEM16_1	2.50	-.25	.47	-.19	.43
STEM16_2	4.20	.60	.32	.50	.24
STEM16_3	4.08	.54	.35	.50	.20
STEM16_4	3.14	.07	.50	.06	.32
STEM17_1	3.30	.15	.49	-.11	.31
STEM17_2	4.01	.50	.37	.44	.18
STEM17_3	4.01	.50	.37	.30	.23
STEM17_4	2.31	-.34	.44	-.34	.38
STEM18_1	3.56	.28	.46	.16	.39
STEM18_2	4.07	.54	.36	.37	.35
STEM18_3	3.88	.44	.40	.35	.01
STEM18_4	3.08	.04	.50	-.13	.40
STEM19_1	4.41	.71	.25	.61	.20
STEM19_2	1.89	-.55	.35	-.40	.21
STEM19_3	2.38	-.31	.45	-.26	.31
STEM19_4	3.49	.25	.47	.09	.36
STEM20_1	3.89	.44	.40	.27	.13
STEM20_2	2.60	-.20	.48	-.30	.26
STEM20_3	3.15	.07	.50	-.11	.32
STEM20_4	3.42	.21	.48	.07	.23
STEM21_1	3.75	.38	.43	.24	.26
STEM21_2	3.18	.09	.50	-.04	.41
STEM21_3	3.57	.28	.46	.15	.06
STEM21_4	3.68	.34	.44	.25	.20
STEM22_1	4.30	.65	.29	.63	.19
STEM22_2	2.60	-.20	.48	-.18	.22
STEM22_3	3.42	.21	.48	.00	.47
STEM22_4	3.10	.05	.50	.01	.40
STEM23_1	3.69	.35	.44	.21	.24
STEM23_2	2.98	-.01	.50	-.15	.40
STEM23_3	3.67	.34	.44	.18	.29
STEM23_4	4.04	.52	.37	.39	.21
STEM24_1	3.63	.31	.45	.12	.36
STEM24_2	2.59	-.20	.48	-.31	.46
STEM24_3	1.76	-.62	.31	-.62	.15
STEM24_4	4.35	.67	.27	.61	.09

STEM25_1	1.96	-.52	.36	-.51	.16
STEM25_2	3.80	.40	.42	.16	.27
STEM25_3	2.24	-.38	.43	-.37	.37
STEM25_4	4.49	.75	.22	.63	.17
STEM26_1	4.00	.50	.37	.31	.24
STEM26_2	3.71	.36	.44	.06	.41
STEM26_3	3.91	.46	.40	.42	.28
STEM26_4	4.42	.71	.25	.65	.15
STEM27_1	4.21	.60	.32	.49	.17
STEM27_2	3.74	.37	.43	.15	.33
STEM27_3	4.02	.51	.37	.41	.25
STEM27_4	2.04	-.48	.39	-.45	.31
STEM28_1	2.19	-.40	.42	-.39	.24
STEM28_2	3.98	.49	.38	.34	.23
STEM28_3	2.53	-.24	.47	-.26	.32
STEM28_4	3.96	.48	.39	.39	.06
STEM29_1	3.55	.27	.46	.25	.35
STEM29_2	3.71	.36	.44	.27	.34
STEM29_3	4.42	.71	.25	.61	.18
STEM29_4	3.88	.44	.40	.30	.23
STEM30_1	2.48	-.26	.47	-.20	.22
STEM30_2	3.12	.06	.50	-.07	.24
STEM30_3	4.17	.58	.33	.47	.15
STEM30_4	3.81	.41	.42	.27	.26

Note. Mean: Mean item scores, H_{EI}: hypothetical loadings on Emotional Intelligence Component, H_{AR}: hypothetical loadings on Acquiescent Responding Component, EI: observed loadings on Emotional Intelligence Component, AR: observed loadings on Acquiescent Responding Component.

Table 9

Mean Item Scores, Hypothetical Loadings, and Observed Loadings after Orthogonal Procrustes Rotation of STEU-R in a Two-Componential Structure

Item	Mean	H_{EI}	H_{AR}	EI	AR
Item1_1	4.05	.02	.50	-.15	.42
Item2_1	6.64	.88	.11	.49	.21
Item1_2	6.37	.79	.19	.50	.14
Item2_2	1.74	-.75	.22	-.44	.22
Item1_3	5.40	.47	.39	.22	.19
Item2_3	4.60	.20	.48	.04	.34
Item1_4	5.35	.45	.40	.06	.42
Item2_4	3.61	-.13	.49	-.22	.54
Item1_5	6.06	.69	.26	.49	.14
Item2_5	1.74	-.75	.22	-.62	.24
Item1_6	6.21	.74	.23	.50	.14
Item2_6	2.64	-.45	.40	-.36	.41
Item1_7	3.65	-.12	.49	-.09	.17
Item2_7	6.02	.67	.27	.38	.08
Item1_8	4.61	.20	.48	.05	.37
Item2_8	2.87	-.38	.43	-.24	.46
Item1_9	2.23	-.59	.33	-.37	.33
Item2_9	2.12	-.63	.30	-.32	.31
Item1_10	2.32	-.56	.34	-.31	.40
Item2_10	6.15	.72	.24	.47	.00
Item1_11	5.37	.46	.40	.30	.36
Item2_11	4.28	.09	.50	-.01	.24
Item1_12	4.49	.16	.49	-.02	.39
Item2_12	6.25	.75	.22	.39	.10
Item1_13	6.56	.85	.14	.41	.13
Item2_13	1.86	-.71	.25	-.37	.30
Item1_14	6.61	.87	.12	.45	.18
Item2_14	6.76	.92	.08	.49	.11
Item1_15	2.27	-.58	.33	-.43	.28
Item2_15	6.23	.74	.22	.45	-.08
Item1_16	1.48	-.84	.15	-.63	.21
Item2_16	6.46	.82	.16	.55	.12
Item1_17	4.45	.15	.49	.09	.37
Item2_17	1.61	-.80	.18	-.39	.30
Item1_18	6.63	.88	.12	.54	.12
Item2_18	6.40	.80	.18	.53	.16
Item1_19	3.86	-.05	.50	-.07	.12
Item2_19	4.67	.22	.47	.09	.17
Item1_20	6.43	.81	.17	.49	.10
Item2_20	2.40	-.53	.36	-.40	.22
Item1_21	1.71	-.76	.21	-.56	.22
Item2_21	6.40	.80	.18	.49	.12
Item1_22	2.18	-.61	.32	-.45	.25
Item2_22	2.07	-.64	.29	-.44	.25
Item1_23	3.08	-.31	.45	-.25	.45
Item2_23	4.79	.26	.47	.09	.46

Item1_24	1.58	-.81	.18	-.52	.27
Item2_24	6.28	.76	.21	.55	.12
Item1_25	3.99	.00	.50	-.04	.45
Item2_25	3.97	-.01	.50	.02	.39
Item1_26	3.17	-.28	.46	-.21	.39
Item2_26	4.53	.18	.48	.09	.44
Item1_27	3.82	-.06	.50	-.06	.35
Item2_27	3.06	-.31	.45	-.28	.44
Item1_28	5.30	.43	.41	.10	.26
Item2_28	6.46	.82	.16	.47	.14
Item1_29	4.72	.24	.47	.11	.10
Item2_29	4.42	.14	.49	.12	.23
Item1_30	6.77	.92	.07	.40	.23
Item2_30	6.77	.92	.07	.40	.20

Note. Mean: Mean item scores, H_{EI}: hypothetical loadings on Emotional Intelligence Component, H_{AR}: hypothetical loadings on Acquiescent Responding Component, EI: observed loadings on Emotional Intelligence Component, AR: observed loadings on Acquiescent Responding Component.

Table 11
Mean Item Scores, Hypothetical Loadings, and Observed Loadings after Orthogonal Procrustes Rotation of STEM-R in a Two-Componential Structure

Item	Mean	H_{EI}	H_{AR}	EI	AR
Item1_1	3.32	-.23	.47	-.26	.31
Item2_1	3.55	-.15	.49	-.22	.47
Item1_2	4.77	.26	.47	-.06	.32
Item2_2	5.53	.51	.37	.44	.21
Item1_3	5.49	.50	.38	.48	.17
Item2_3	4.89	.30	.46	.00	.30
Item1_4	2.81	-.40	.42	-.27	.35
Item2_4	6.06	.69	.27	.62	.19
Item1_5	5.69	.56	.34	.57	.08
Item2_5	2.70	-.43	.41	-.50	.25
Item1_6	3.71	-.10	.50	-.18	.46
Item2_6	4.39	.13	.49	.07	-.09
Item1_7	5.30	.43	.41	.16	.26
Item2_7	5.90	.63	.30	.56	.16
Item1_8	5.98	.66	.28	.55	.22
Item2_8	5.63	.54	.35	.41	.11
Item1_9	5.99	.66	.28	.63	.20
Item2_9	2.76	-.41	.41	-.37	.34
Item1_10	2.87	-.38	.43	-.34	.28
Item2_10	5.42	.47	.39	.25	.34
Item1_11	3.05	-.32	.45	-.35	.35
Item2_11	6.11	.70	.25	.69	.17
Item1_12	3.03	-.32	.45	-.35	.24
Item2_12	6.12	.71	.25	.50	.12
Item1_13	4.29	.10	.50	-.03	.12
Item2_13	3.12	-.29	.46	-.22	.22
Item1_14	5.99	.66	.28	.55	.24
Item2_14	2.54	-.49	.38	-.42	.21
Item1_15	5.67	.56	.34	.51	.16
Item2_15	4.15	.05	.50	-.11	.26
Item1_16	5.58	.53	.36	.45	.21
Item2_16	4.09	.03	.50	.02	.39
Item1_17	5.82	.61	.32	.56	.12
Item2_17	3.22	-.26	.47	-.38	.36
Item1_18	4.53	.18	.48	.04	.24
Item2_18	5.61	.54	.36	.51	.16
Item1_19	3.48	-.17	.49	.03	.28
Item2_19	3.41	-.20	.48	-.38	.24
Item1_20	3.82	-.06	.50	-.10	.35
Item2_20	4.91	.30	.45	.06	.29
Item1_21	4.12	.04	.50	.00	.13
Item2_21	5.21	.40	.42	.31	.22
Item1_22	6.01	.67	.28	.59	.13
Item2_22	4.16	.05	.50	-.02	.30
Item1_23	4.31	.10	.49	-.01	.37
Item2_23	5.39	.46	.39	.39	.14

Item1_24	3.50	-.17	.49	-.28	.45
Item2_24	2.00	-.67	.28	-.54	.32
Item1_25	2.72	-.43	.41	-.20	.32
Item2_25	2.95	-.35	.44	-.32	.23
Item1_26	5.25	.42	.41	.24	.15
Item2_26	6.27	.76	.21	.58	.20
Item1_27	5.93	.64	.29	.63	.21
Item2_27	2.61	-.46	.39	-.42	.27
Item1_28	3.13	-.29	.46	-.14	.35
Item2_28	3.70	-.10	.50	-.30	.38
Item1_29	6.17	.72	.24	.52	.20
Item2_29	5.11	.37	.43	.31	.21
Item1_30	3.99	.00	.50	.03	.35
Item2_30	5.78	.59	.32	.49	.26

Note. Mean: Mean item scores, H_{EI}: hypothetical loadings on Emotional Intelligence Component, H_{AR}: hypothetical loadings on Acquiescent Responding Component, EI: observed loadings on Emotional Intelligence Component, AR: observed loadings on Acquiescent Responding Component.

Table 11

Mean Item Scores, Hypothetical Loadings, and Observed Loadings after Orthogonal Procrustes Rotation of GERT-R in a Two-Componential Structure

Item	Mean	H_{EI}	H_{AR}	EI	AR
Item1	5.01	.34	.44	.10	.34
Item2	4.95	.32	.45	.16	.38
Item3	3.65	-.12	.49	-.12	.50
Item4	4.37	.12	.49	-.09	.50
Item5	4.93	.31	.45	.08	.51
Item6	3.62	-.13	.49	-.13	.25
Item7	2.15	-.62	.31	-.45	.47
Item8	5.00	.33	.44	-.02	.52
Item9	3.53	-.16	.49	-.14	.51
Item10	1.51	-.83	.16	-.65	.27
Item11	6.04	.68	.27	.43	.22
Item12	2.90	-.37	.43	-.29	.47
Item13	5.94	.65	.29	.23	.41
Item14	1.82	-.73	.23	-.50	.34
Item15	6.46	.82	.16	.51	.17
Item16	1.40	-.87	.12	-.68	.17
Item17	5.97	.66	.28	.28	.36
Item18	1.77	-.74	.22	-.59	.33
Item19	2.63	-.46	.40	-.32	.32
Item20	6.83	.94	.05	.39	.15
Item21	1.35	-.88	.11	-.61	.20
Item22	4.05	.02	.50	-.06	.54
Item23	1.74	-.75	.22	-.58	.32
Item24	2.31	-.56	.34	-.40	.39
Item25	2.18	-.61	.32	-.46	.39
Item26	5.41	.47	.39	.21	.29
Item27	1.45	-.85	.14	-.70	.25
Item28	6.45	.82	.17	.39	.10
Item29	4.04	.01	.50	-.13	.51
Item30	5.53	.51	.37	.12	.45
Item31	2.02	-.66	.28	-.53	.33
Item32	2.87	-.38	.43	-.27	.44
Item33	2.73	-.42	.41	-.25	.44
Item34	3.72	-.09	.50	-.07	.43
Item35	1.35	-.88	.11	-.65	.18
Item36	2.76	-.41	.41	-.32	.47
Item37	1.40	-.87	.12	-.61	.22
Item38	6.46	.82	.16	.36	.13
Item39	4.14	.05	.50	-.03	.49
Item40	5.77	.59	.33	.30	.27
Item41	1.61	-.80	.18	-.64	.29
Item42	1.29	-.90	.09	-.63	.11
Item43	3.12	-.29	.46	-.22	.50
Item44	3.04	-.32	.45	-.19	.51
Item45	2.39	-.54	.36	-.37	.43
Item46	3.79	-.07	.50	-.08	.33

Item47	4.02	.01	.50	-.10	.41
Item48	1.57	-.81	.17	-.60	.25
Item49	6.72	.91	.09	.44	.19
Item50	6.66	.89	.11	.41	.16
Item51	6.32	.77	.20	.44	.12
Item52	6.65	.88	.11	.57	.13
Item53	1.50	-.83	.15	-.65	.23
Item54	2.11	-.63	.30	-.34	.27
Item55	1.35	-.88	.11	-.64	.20
Item56	5.53	.51	.37	.25	.39
Item57	1.29	-.90	.09	-.69	.10
Item58	1.31	-.90	.10	-.66	.22
Item59	5.18	.39	.42	.15	.35
Item60	6.19	.73	.23	.46	.22
Item61	5.86	.62	.31	.20	.28
Item62	6.14	.71	.24	.33	.17
Item63	4.68	.23	.47	-.02	.51
Item64	5.75	.58	.33	.25	.13
Item65	5.87	.62	.31	.30	.23
Item66	1.44	-.85	.14	-.64	.22
Item67	1.87	-.71	.25	-.58	.35
Item68	6.50	.83	.15	.49	.23
Item69	3.48	-.17	.48	-.18	.48
Item70	5.07	.36	.44	.15	.32

Note. Mean: Mean item scores, H_{EI}: hypothetical loadings on Emotional Intelligence Component, H_{AR}: hypothetical loadings on Acquiescent Responding Component, EI: observed loadings on Emotional Intelligence Component, AR: observed loadings on Acquiescent Responding Component.

Table 12

Item Means and Correlations of Emotional Intelligence Scores (EIS) and Acquiescent Responding Scores (ARS) with Proportion of Observed Extreme Responses per EI Test

EI test	Item Mean	r_{EIS}	r_{ARS}
MSCEIT Faces	1.96	.73	-.30
MSCEIT Pictures	1.89	.51	-.38
MSCEIT Facilitation	2.54	.70	-.15
MSCEIT Sensations	2.81	.72	-.02
MSCEIT Managing Emotions	3.21	.72	.11
MSCEIT Emotional Relationships	2.91	.73	-.04
STEU	3.35	.68	-.18
STEM	3.47	.71	.09
STEU-R[a]	3.27	.73	-.01
STEM-R[a]	3.33	.59	.14
GERT-R[a]	2.87	.67	-.11

Note. [a] Item mean has been rescaled from 1 to 5 for comparability with the other EI tests.

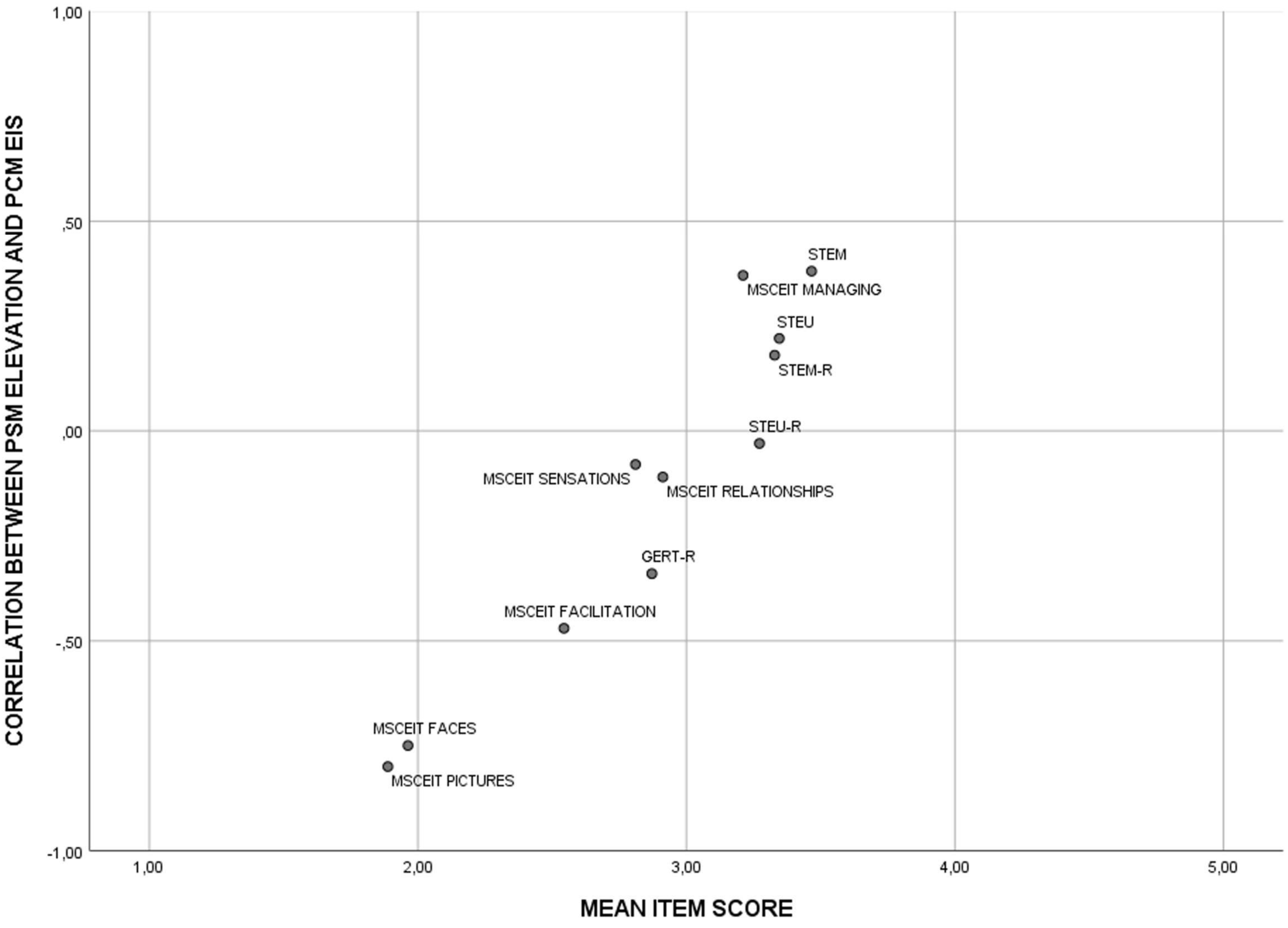

Figure 1. Variation of the Correlation between PSM Elevation and PCM Emotional Intelligence Scores (EIS) as a Function of the Mean Item Score Across All Items

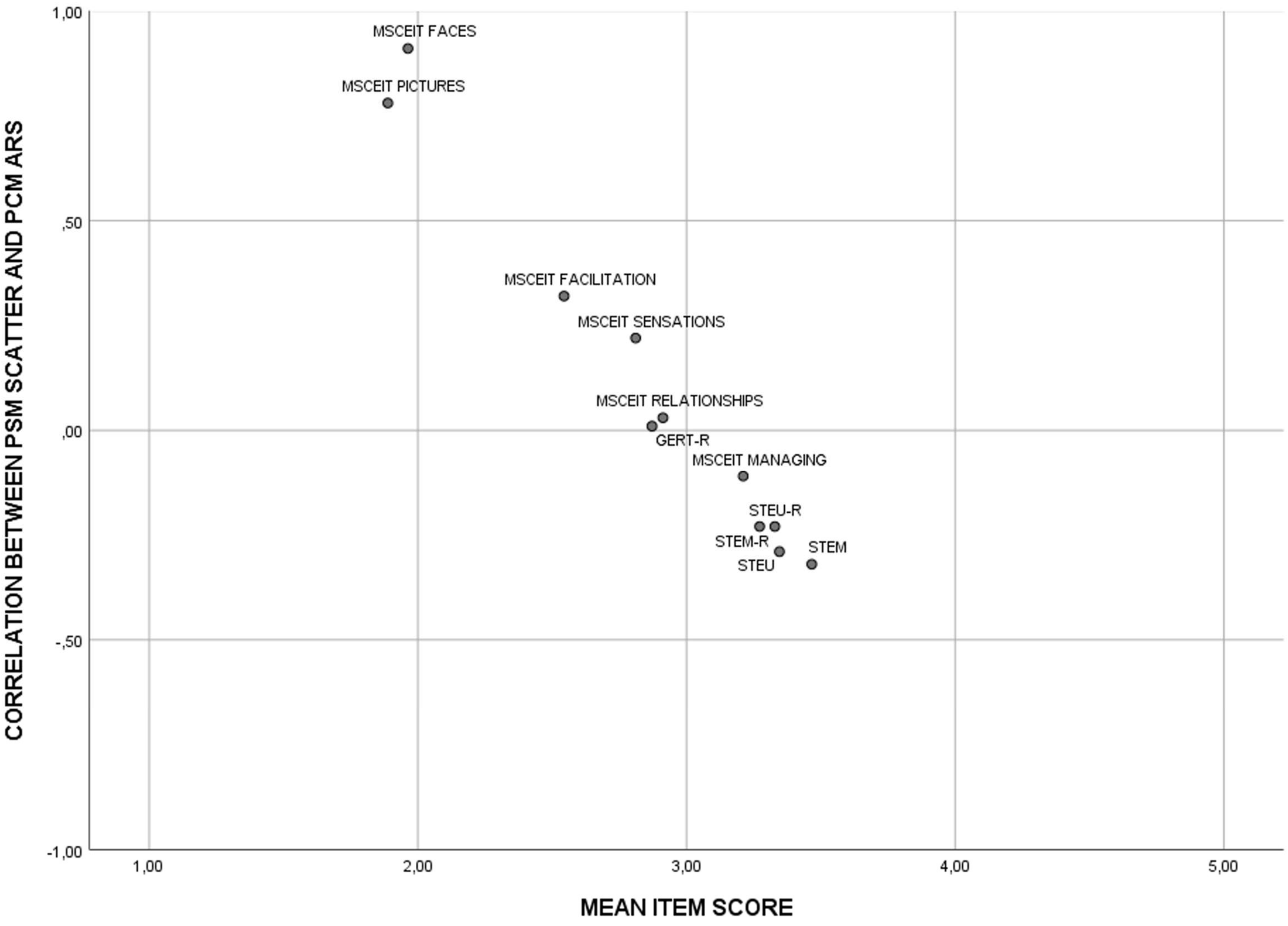

Figure 2. Variation of the Correlation between PSM Scatter and PCM Acquiescent Responding Scores (ARS) as a Function of the Mean Item Score Across All Items
